# Supplementary material for: GWAS on short tandem repeats identifies genetic mechanisms in Alzheimer’s disease
Source: Nat Commun. 2026 Jun 4;17:4968. doi: 10.1038/s41467-026-73902-7 (PMC13237113; doi:10.1038/s41467-026-73902-7)
Supplement: Supplementary file 1 — Supplementary Information [file 41467_2026_73902_MOESM1_ESM.pdf]

GWAS on short tandem repeats identifies novel genetic mechanisms in Alzheimer's disease: *Supplementary Information*

David Gmelin[1], Olena Ohlei[1,2], M. Muaaz Aslam[1], Marit P. Junge[1], Laura Parkkinen[3], Kristina Mullin[4], Dmitry Prokopenko[4,5], Christina M. Lill[2,6], Rudolph E. Tanzi[4,5], Valerija Dobricic[1, †] and Lars Bertram[1, †, *]

[1] Lübeck Interdisciplinary Platform for Genome Analytics (LIGA), University of Lübeck, Lübeck, Germany.
[2] Institute of Epidemiology and Social Medicine, University of Münster, Münster, Germany
[3] Nuffield Department of Clinical Neurosciences, University of Oxford, Oxford, UK
[4] Genetics and Aging Research Unit and McCance Center for Brain Health, Department of Neurology, Massachusetts General Hospital, Boston, MA, USA.
[5] Harvard Medical School, Boston, MA, USA
[6] Ageing Epidemiology Research Unit (AGE), School of Public Health, Imperial College London, London, UK

† These authors contributed equally: Valerija Dobricic, Lars Bertram
*Corresponding author, e-mail: lars.bertram@uni-luebeck.de

Supplementary Table 1. Number of AD cases, AD-by-proxy cases, and controls in different self-reported ethnic groups extracted from the UKB.

Ethnic group	AD-case	AD-by-proxy cases	Controls	Total	Female	Male	Age (SD)
White-British	2947	42923	249681	295551	164209	131342	56.6 (7.94)
Other-White	185	2803	17852	20840	12317	8523	54.8 (8.23)
Asian	49	373	5772	6194	3000	3194	53 (8.42)
Black	50	321	3969	4340	2553	1787	51.3 (7.77)
Other	22	223	2673	2918	1704	1214	52.3 (7.93)
Mixed	10	196	1388	1594	1040	554	51.7 (7.89)
Chinese	4	89	966	1059	678	381	51.8 (7.53)
No-answer	20	120	810	950	429	521	57.3 (7.94)
Total	3287	47048	283111	333446	185930	147516	56.3 (8.03)

Supplementary Table 2. Number of AD cases, AD-by-proxy cases, and controls in different self-reported ethnic groups from the UKB with available WGS-based STR data.

Ethnic group	AD-case	AD-by-proxy cases	Controls	Total	Female	Male	Age (SD)
White-British	928	13759	80514	95201	53811	41390	56.5 (7.98)
Other-White	48	887	5632	6567	3955	2612	54.8 (8.23)
Asian	13	126	1949	2088	1009	1079	53 (8.39)
Black	17	88	1213	1318	790	528	51.2 (7.73)
Other	8	85	840	933	565	368	52.4 (8.04)
Mixed	2	68	460	530	352	178	51.7 (7.67)
Chinese	2	33	280	315	199	116	51.6 (7.84)
No-answer	8	43	286	337	160	177	57.2 (7.84)
Total	1026	15089	91174	107289	60841	46448	56.2 (8.06)

Supplementary Table 3. Heritability estimates using the GCTA-LDMS method across 17 sample batches (~15,000 individuals each).

Batch	h(SNP+STR)	h(SNP)	h(STR)	STR contribution*
1	0.3801	0.3713	0.3104	0.02327
2	0.3434	0.3452	0.2827	-0.00517
3	0.4292	0.4133	0.3378	0.03713
4	0.4085	0.3845	0.3496	0.05869
5	0.4076	0.3873	0.3283	0.04981
6	0.3523	0.3300	0.2908	0.06352
7	0.3889	0.3683	0.3224	0.05295
8	0.3585	0.3469	0.2739	0.03229
9	0.3279	0.3322	0.2548	-0.01307
10	0.3855	0.3931	0.3071	-0.01952
11	0.4386	0.4282	0.3522	0.02382
12	0.3590	0.3629	0.2873	-0.01081
13	0.3818	0.3556	0.3157	0.06864
14	0.3941	0.3694	0.3225	0.06279
15	0.3946	0.3954	0.3247	-0.00193
16	0.3611	0.3556	0.2736	0.01534
17	0.3634	0.3602	0.2983	0.00893
Average	0.3809	0.3705	0.3078	0.02628

* STR Contribution was calculated as the relative change using the following formula: (h(SNP+STR)-h(SNP))/h(SNP+STR).

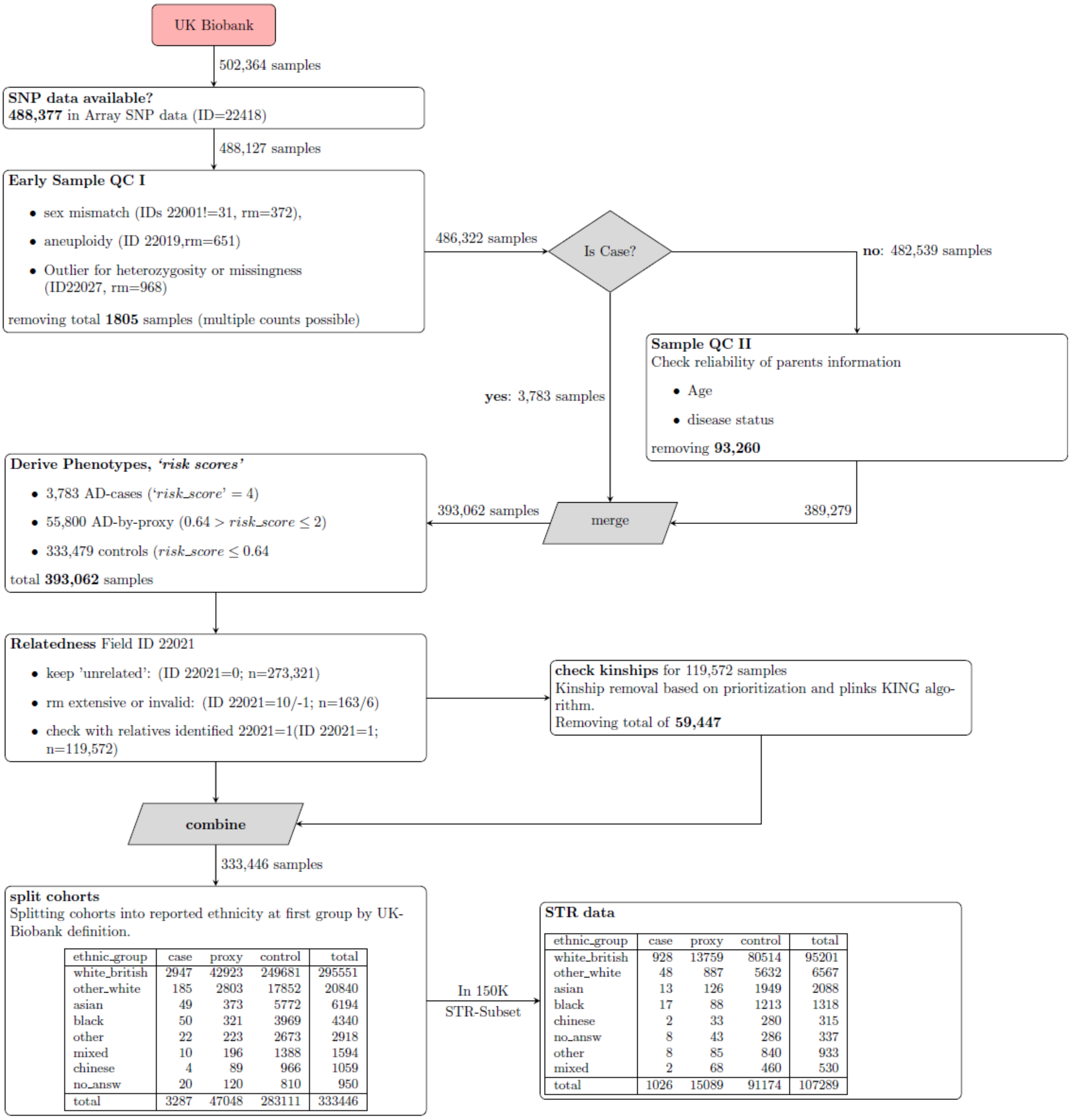

ethnic_group	case	proxy	control	total
white_british	2947	42923	249681	295551
other_white	185	2803	17852	20840
asian	49	373	5772	6194
black	50	321	3969	4340
other	22	223	2673	2918
mixed	10	196	1388	1594
chinese	4	89	966	1059
no_answ	20	120	810	950
total	3287	47048	283111	333446

ethnic_group	case	proxy	control	total
white_british	928	13759	80514	95201
other_white	48	887	5632	6567
asian	13	126	1949	2088
black	17	88	1213	1318
chinese	2	33	280	315
no_answ	8	43	286	337
other	8	85	840	933
mixed	2	68	460	530
total	1026	15089	91174	107289

Supplementary Figure 1. Flowchart of data extraction and filtering implemented to identify eligible sample sets.

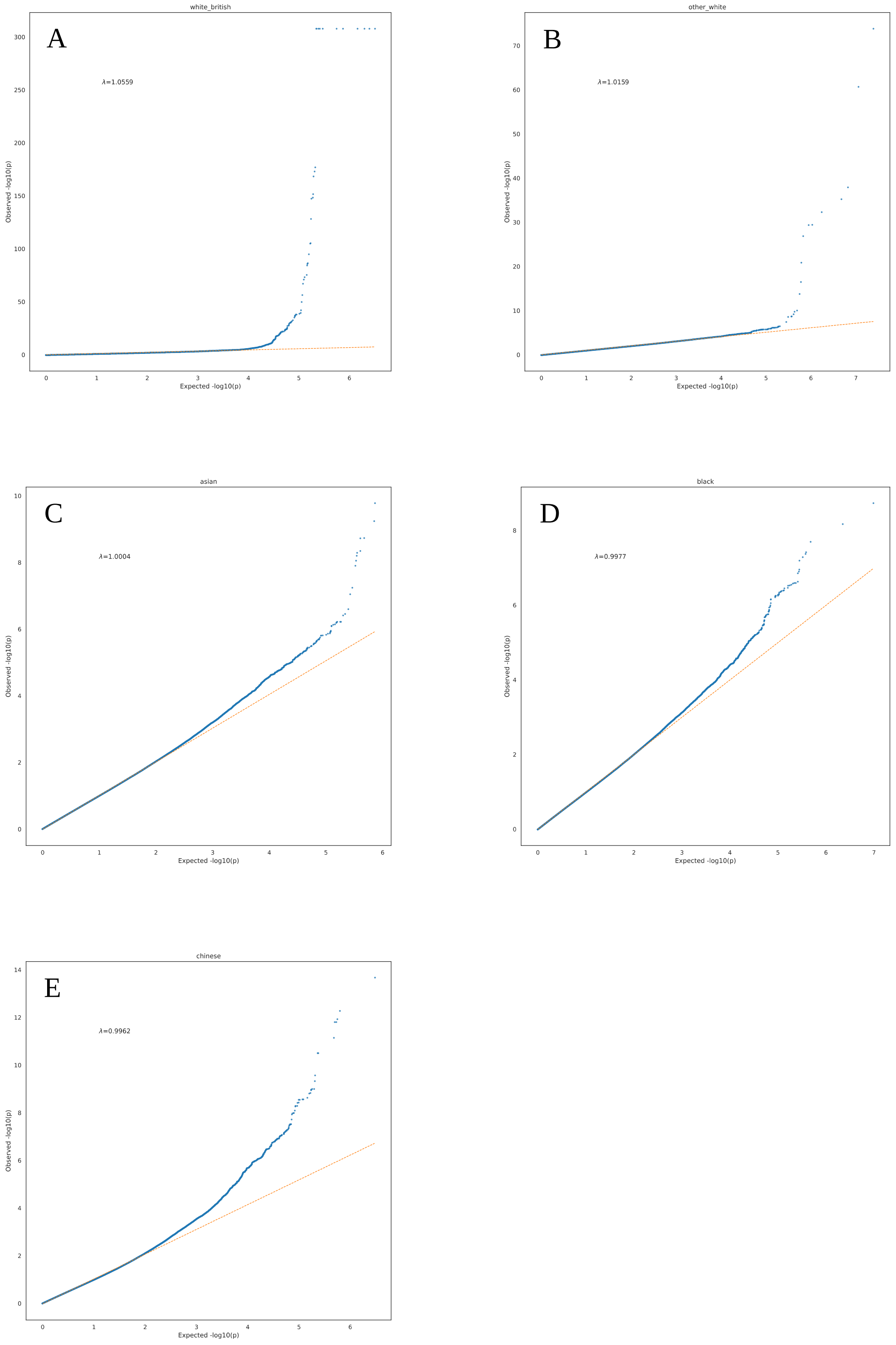

Supplementary Figure 2. QQ plots of imputed STR association results per cohort. The expected –log10 transformed two-sided P values are displayed on the x-axis, while the observed –log10 transformed P values are displayed on the y-axis. A - "White-British", λ=1.0559, n= 295,551; B - "other-White", λ=1.0159, n= 20,840; C - "Asian", λ=1.0004, n= 6,194; D - "Black", λ= 0.9977, n= 4,340; E - "Chinese", λ= 0.9962, n=1,059.

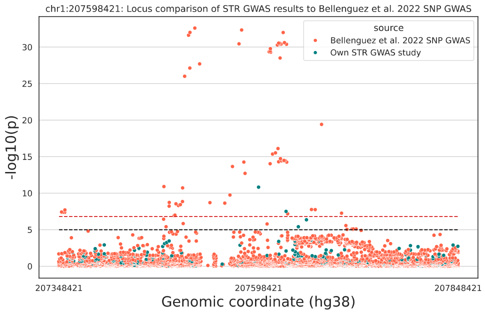
chr1:207598421: Locus comparison of STR GWAS results to Bellenguez et al. 2022 SNP GWAS
source
Bellenguez et al. 2022 SNP GWAS
Own STR GWAS study
-log10(p)
207348421
207598421
207848421
Genomic coordinate (hg38)

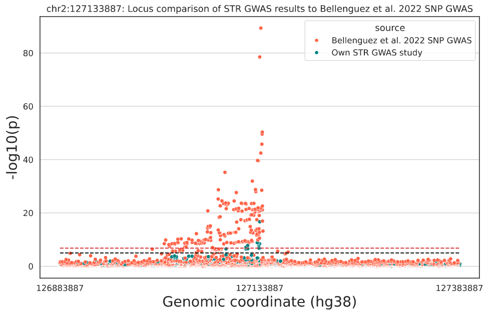
chr2:127133887: Locus comparison of STR GWAS results to Bellenguez et al. 2022 SNP GWAS
source
Bellenguez et al. 2022 SNP GWAS
Own STR GWAS study
-log10(p)
126883887
127133887
127383887
Genomic coordinate (hg38)

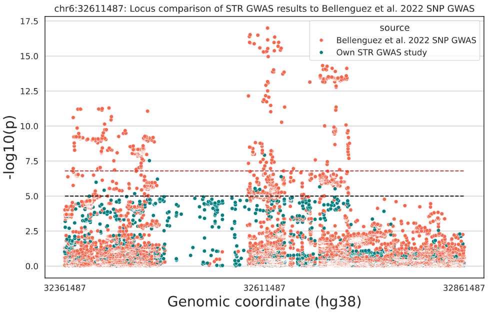
chr6:32611487: Locus comparison of STR GWAS results to Bellenguez et al. 2022 SNP GWAS
source
Bellenguez et al. 2022 SNP GWAS
Own STR GWAS study
-log10(p)
32361487
32611487
32861487
Genomic coordinate (hg38)

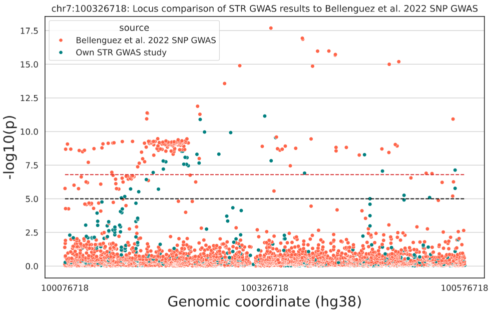
chr7:100326718: Locus comparison of STR GWAS results to Bellenguez et al. 2022 SNP GWAS
source
Bellenguez et al. 2022 SNP GWAS
Own STR GWAS study
-log10(p)
100076718
100326718
100576718
Genomic coordinate (hg38)

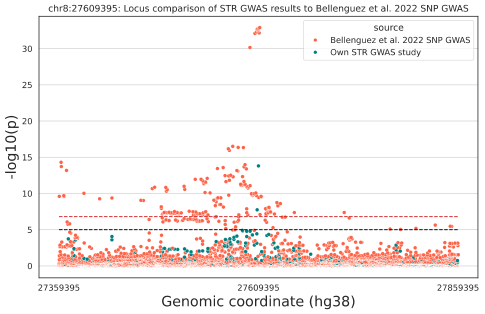
chr8:27609395: Locus comparison of STR GWAS results to Bellenguez et al. 2022 SNP GWAS
source
Bellenguez et al. 2022 SNP GWAS
Own STR GWAS study
-log10(p)
27359395
27609395
27859395
Genomic coordinate (hg38)

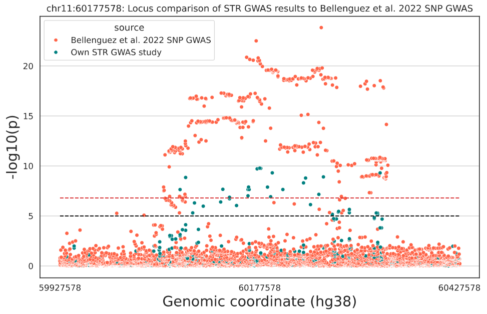
chr11:60177578: Locus comparison of STR GWAS results to Bellenguez et al. 2022 SNP GWAS
source
Bellenguez et al. 2022 SNP GWAS
Own STR GWAS study
-log10(p)
59927578
60177578
60427578
Genomic coordinate (hg38)

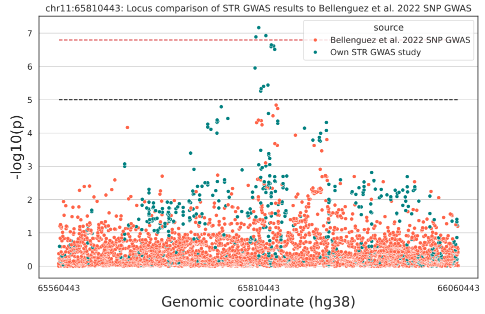
chr11:65810443: Locus comparison of STR GWAS results to Bellenguez et al. 2022 SNP GWAS
source
Bellenguez et al. 2022 SNP GWAS
Own STR GWAS study
-log10(p)
65560443
65810443
66060443
Genomic coordinate (hg38)

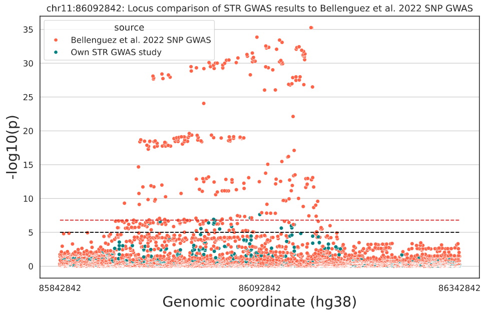
chr11:86092842: Locus comparison of STR GWAS results to Bellenguez et al. 2022 SNP GWAS
source
Bellenguez et al. 2022 SNP GWAS
Own STR GWAS study
-log10(p)
85842842
86092842
86342842
Genomic coordinate (hg38)

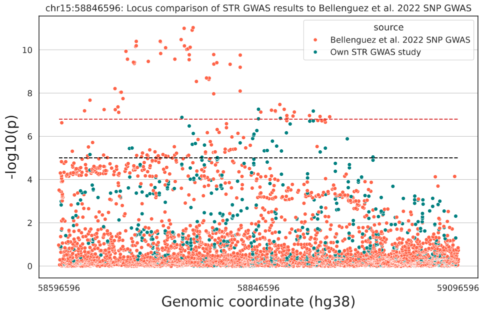
chr15:58846596: Locus comparison of STR GWAS results to Bellenguez et al. 2022 SNP GWAS
source
Bellenguez et al. 2022 SNP GWAS
Own STR GWAS study
-log10(p)
58596596
58846596
59096596
Genomic coordinate (hg38)

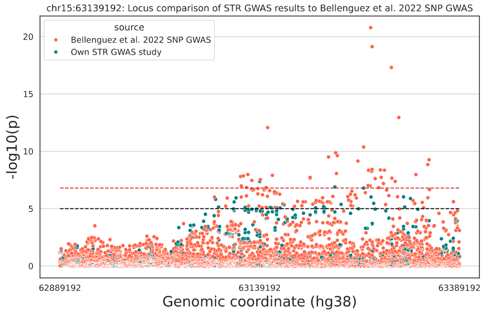
chr15:63139192: Locus comparison of STR GWAS results to Bellenguez et al. 2022 SNP GWAS
source
Bellenguez et al. 2022 SNP GWAS
Own STR GWAS study
-log10(p)
62889192
63139192
63389192
Genomic coordinate (hg38)

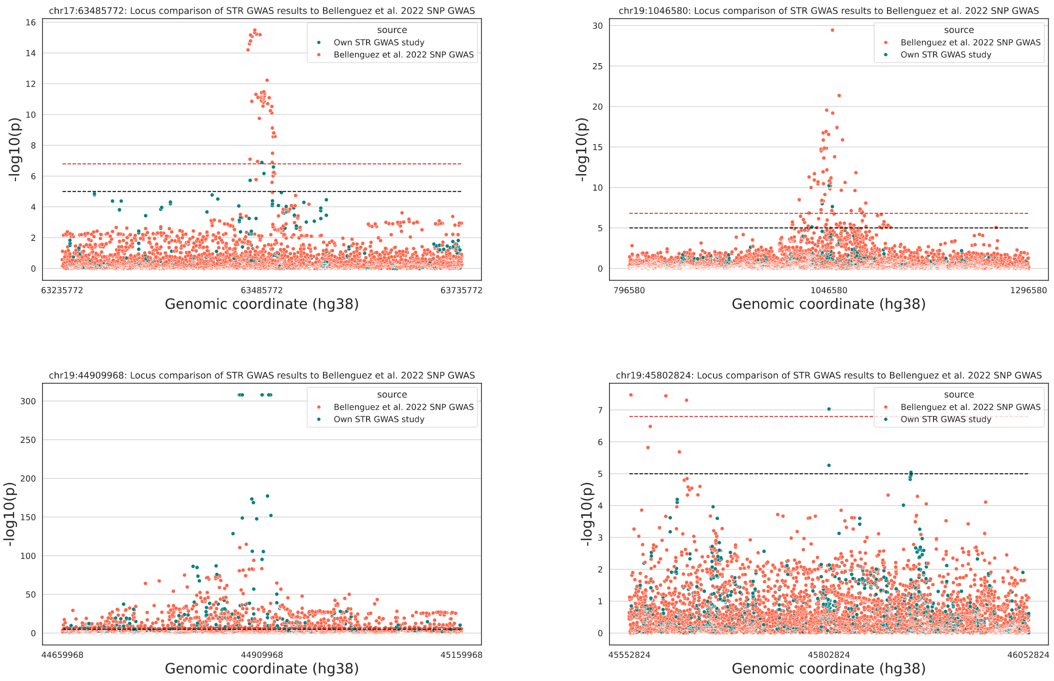

Supplementary Figure 3. Visual comparison of our genome-wide significant STR-GWAS results with those from the SNP-GWAS published by Bellenguez et al.[1]. Our tests were run on the "White-British"-Cohort (n= 295,551) using two-sided linear regression. Red-dashed line represents the Bonferroni-corrected threshold for genome-wide significance (p<1.49E-07). Black-dashed line represents the unadjusted threshold for suggestive significance (p<1.00E-05).

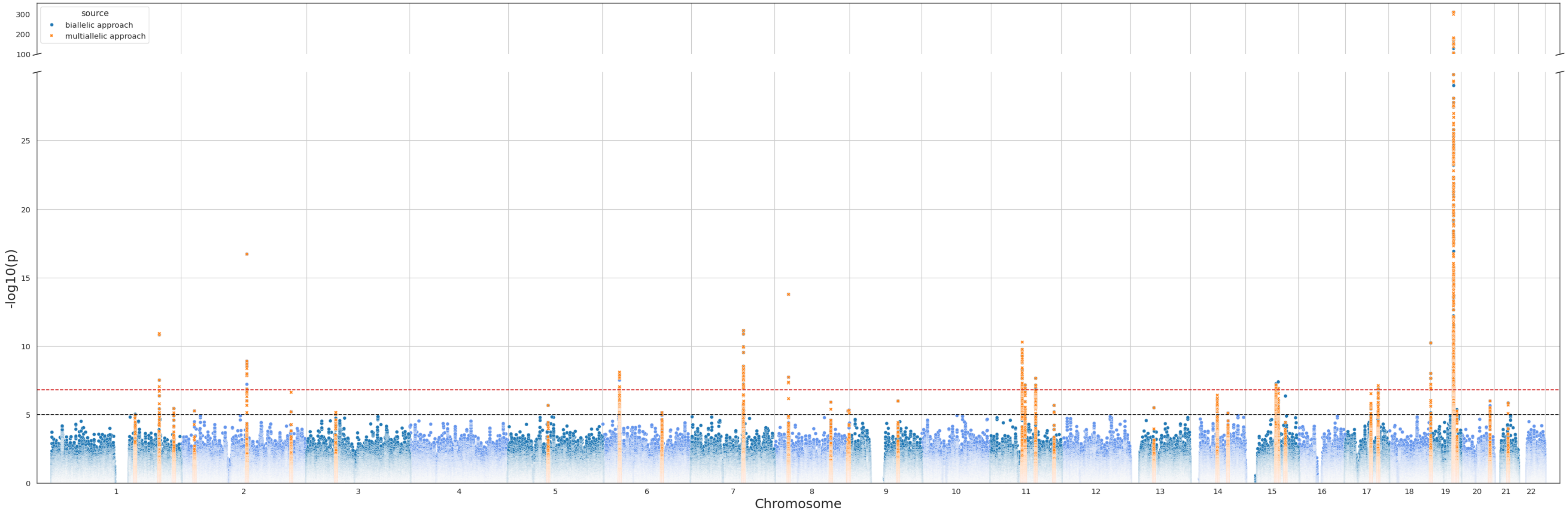

Supplementary Figure 4. Overlay of GWAS results for imputed STRs with bi-allelic (blue) and multi-allelic (orange) approach, for the White-British cohort (n= 295,551). Regression test were run using two-sided linear regression. Red-dashed line represents the Bonferroni-corrected threshold for genome-wide significance (p<1.49E-07). Black-dashed line represents the threshold for suggestive significance (p<1.00E-05, not adjusted for multiple testing). Please note that multi-allelic analyses were limited to variants mapping ±250kb around loci that were at least suggestively significant in the bi-allelic approach. This is due to various notational inconsistencies in the STR imputation catalog (see Methods for more details).

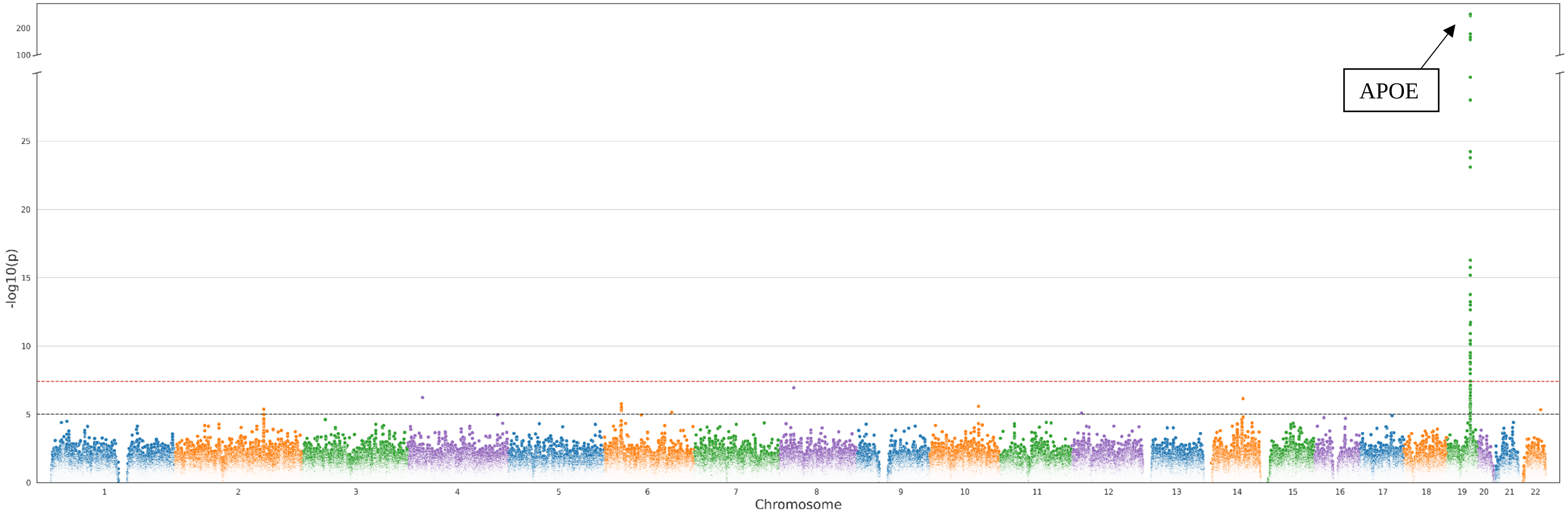

Supplementary Figure 5. Manhattan plot showing results of GWAS using two-sided linear regression for AD and AD-by-proxy status and WGS-derived STRs on "Halldorsson-White-British" cohort (n= 95,201, multi-allelic approach). In the box, the nearest protein-coding gene according to GENCODE V47 is annotated. Horizontal red dashed line indicates the genome-wide significance threshold of 3.77E-08 for these analyses, whereas the black dashed line indicates the suggestive significance threshold of 1.00E-05. Note that y-axis is discontinuous and capped at 300.

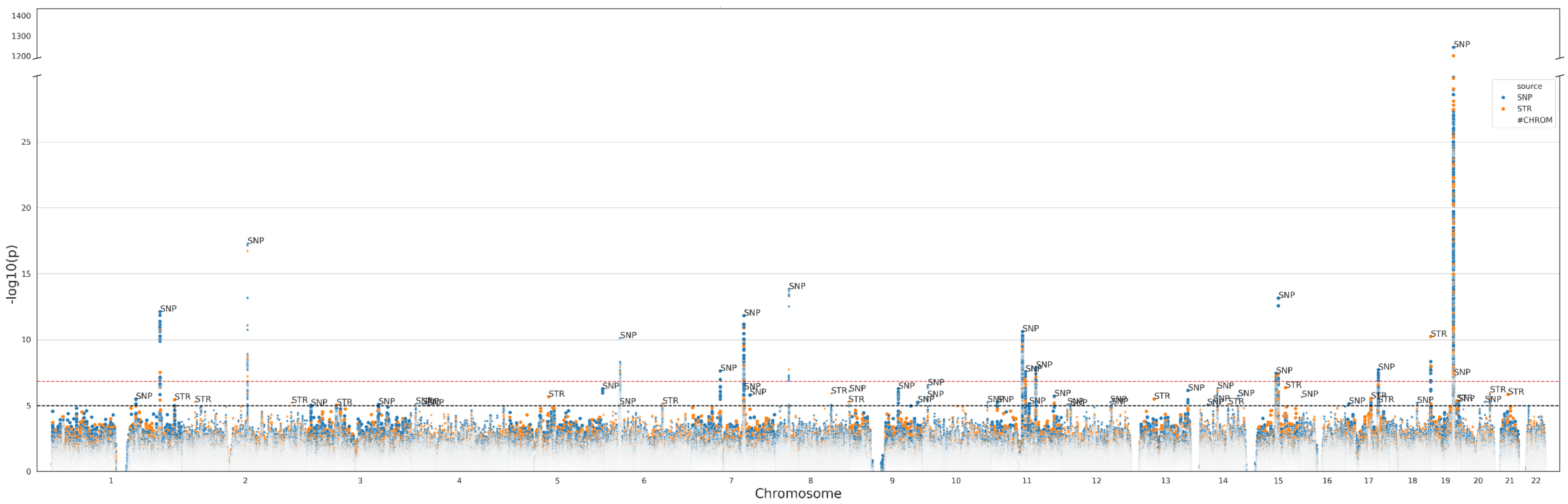

Supplementary Figure 6. Overlay of SNP-based (blue) and STR-based (orange) GWAS results using two-sided linear regression for the White-British cohort (n= 295,551). “SNP” and “STR” labels show the respective lead variant (by P value) in peaks showing genome-wide significant and suggestive evidence for association. Dashed lines represent the thresholds for Bonferroni-corrected genome-wide significance (p<1.49E-07; red) and threshold for suggestive significance that was not adjusted for multiple testing (p<1.00E-05; black).

Supplementary Figures 7 - 19. Results of conditional analysis of genome-wide significant imputed STR loci with corresponding lead SNP using two-sided linear regression on the White-British cohort (n=295,551).

The lead STR-signals were adjusted for the corresponding SNPs at the given locus (upper panel). SNPs utilized for adjustment were the lead SNPs from the largest AD GWAS published to date (Bellenguez et al.[1]), or, if unavailable, the SNPs showing the lowest P value within ±500kb also passing early QC steps performed to prefilter the UKB datasets (i.e. MAF < 0.01 and missing genotyping call rate above 0.02). For the *APOE* region which was excluded in Bellenguez et al.[1], we adjusted for the well-established AD risk allele at rs429358 and SNP rs76320948 from Jansen et al.[2] for the region around the STR-signal at chr19:45802824. Lower panels show the results of reciprocal conditioning of SNP-signals with relevant STR genotypes. Analyses were performed for the White-British cohort (n=295,551).

The horizontal dashed lines indicate the Bonferroni-corrected genome-wide significance threshold (p<1.49E-07; red) and the suggestive significance threshold (p<1.00E-05; black). X-axis: genomic coordinate (hg38), Y-axis: -log10(p) values.

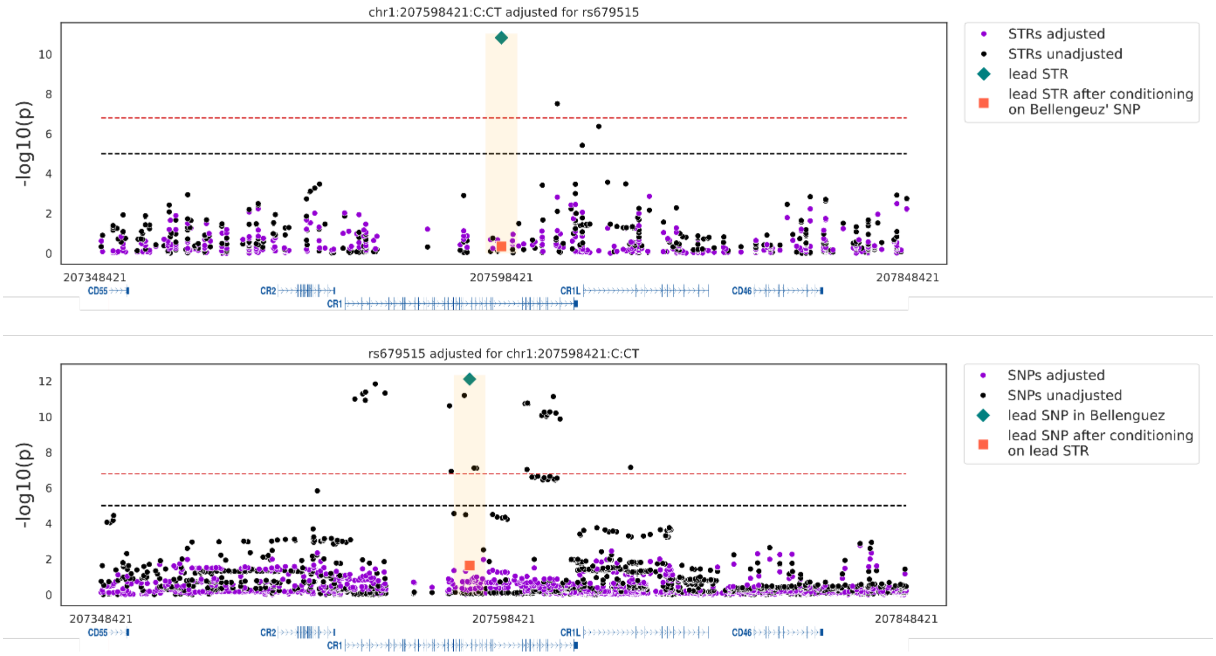

Supplementary Figure 7. STR-SNP pair: chr1:207598421:C:CT - rs679515.

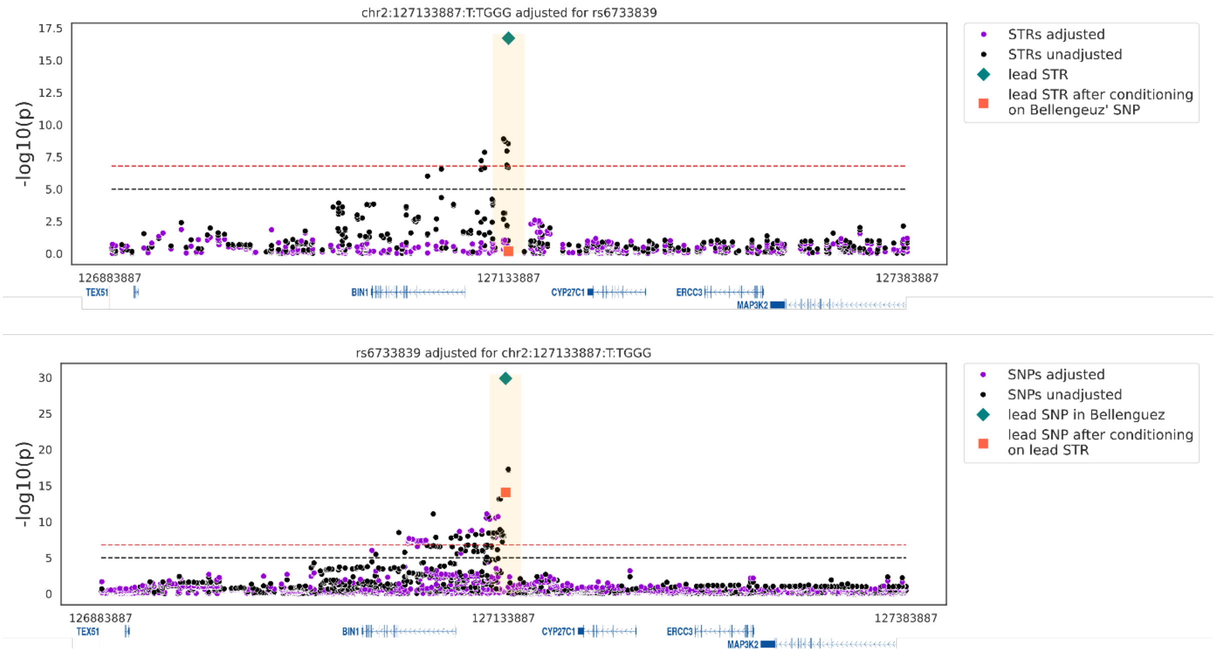

Supplementary Figure 8. STR-SNP pair: chr2:127133887:T:TGGG - rs6733839.

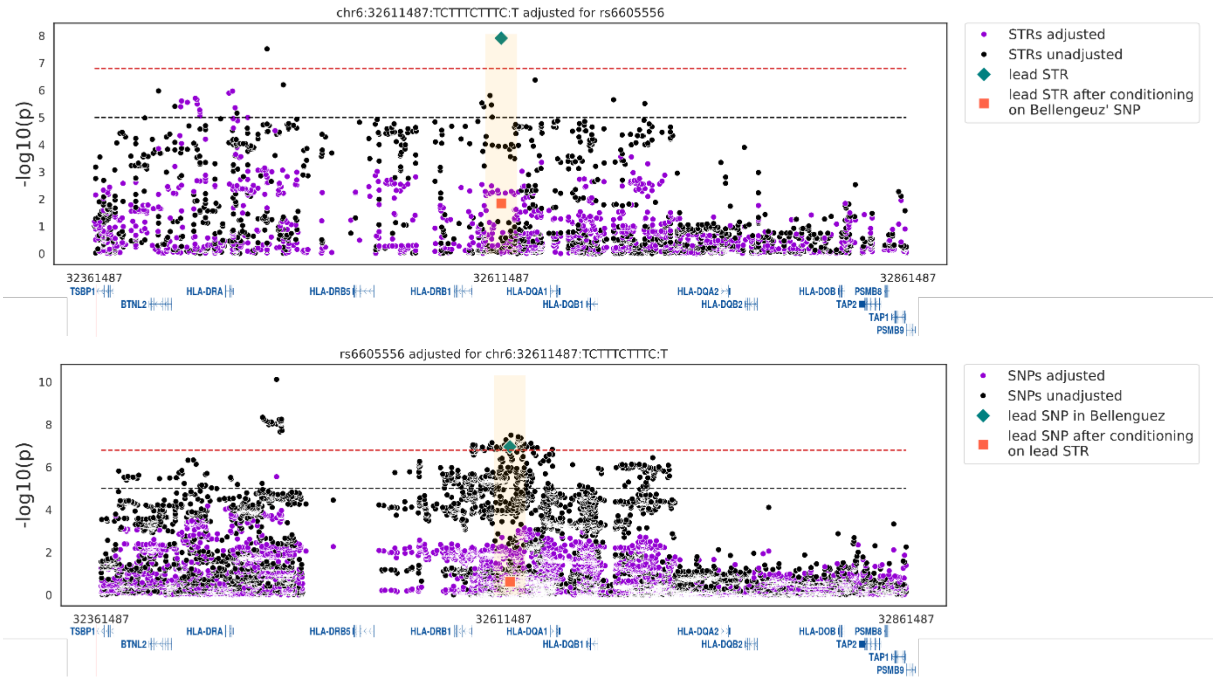

Supplementary Figure 9. STR-SNP pair: chr6:32611487:TCTTTCTTTC:T - rs6605556.

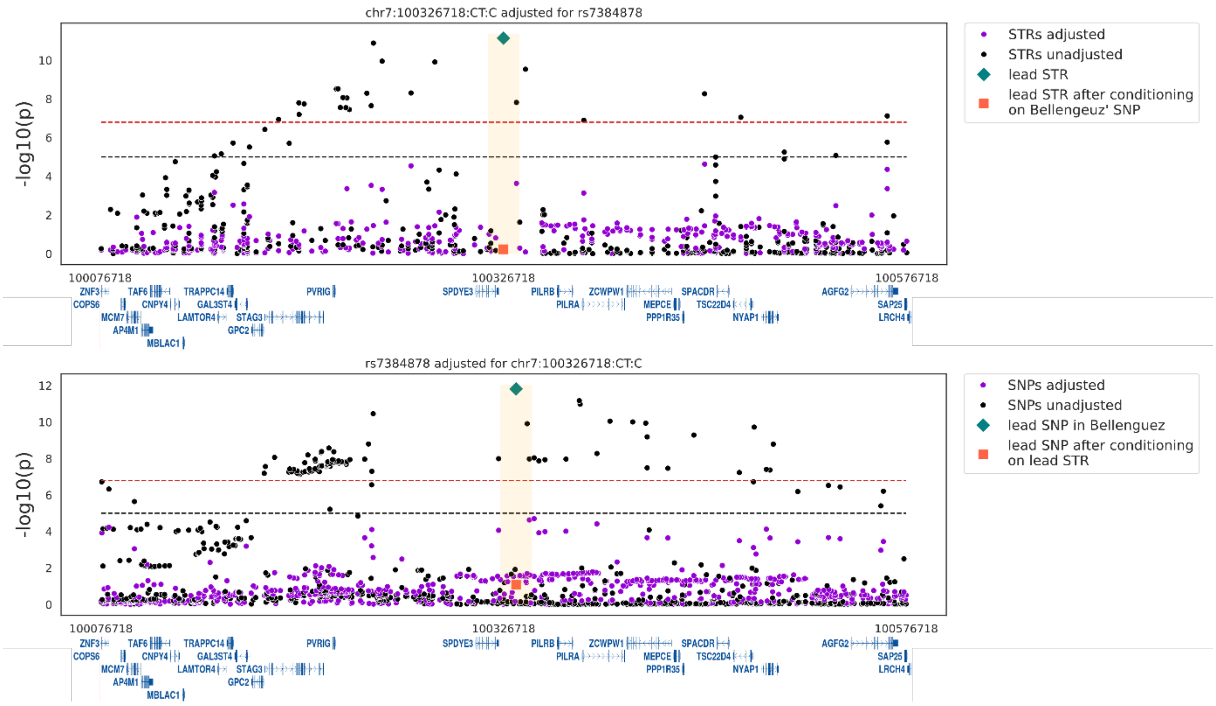

Supplementary Figure 10. STR-SNP pair: chr7:100326718:CT:C - rs7384878.

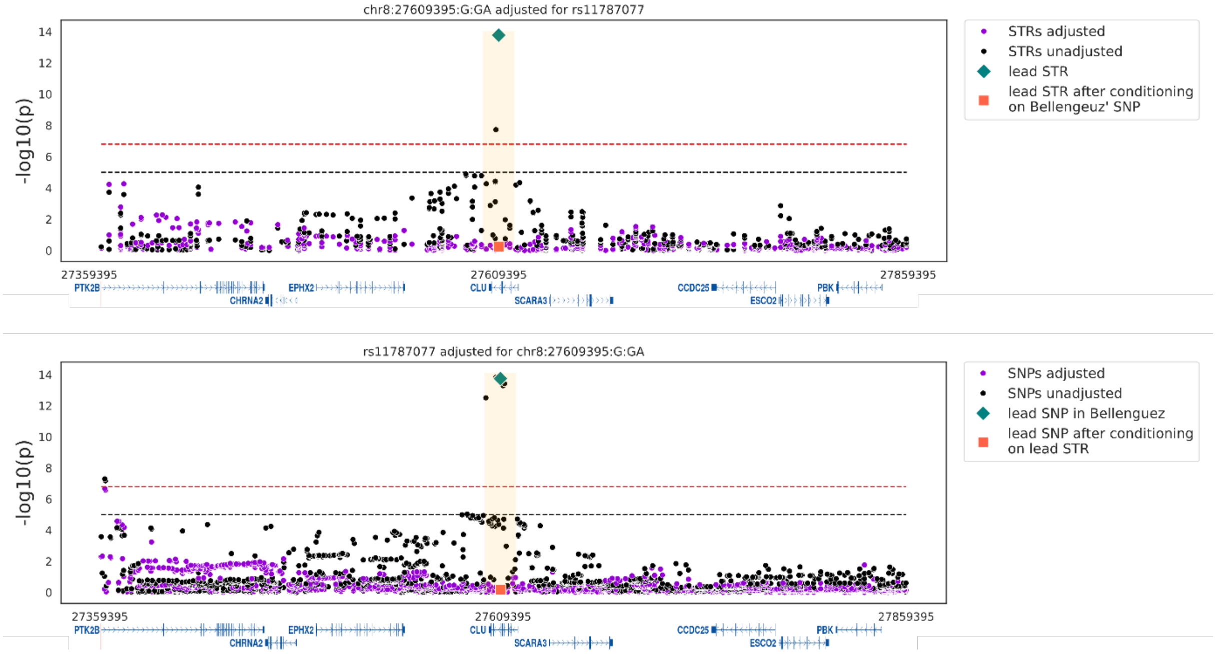

Supplementary Figure 11. STR-SNP pair: chr8:27609395:G:GA - rs11787077.

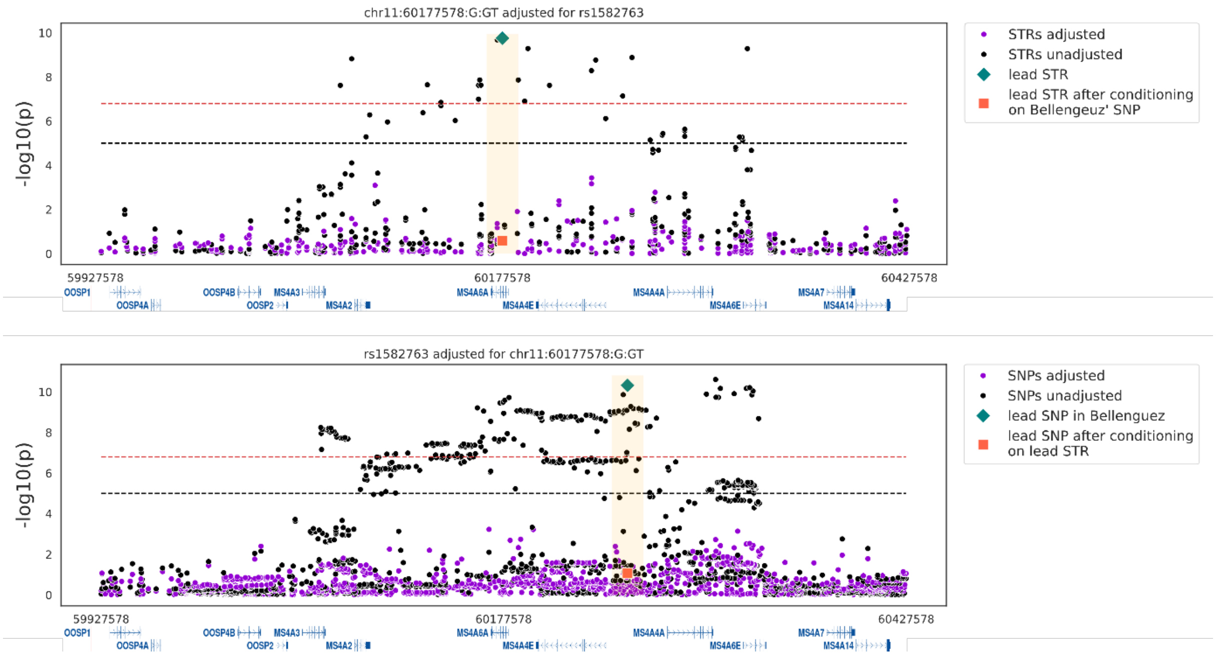

Supplementary Figure 12. STR-SNP pair: chr11:60177578:G:GT - rs1582763.

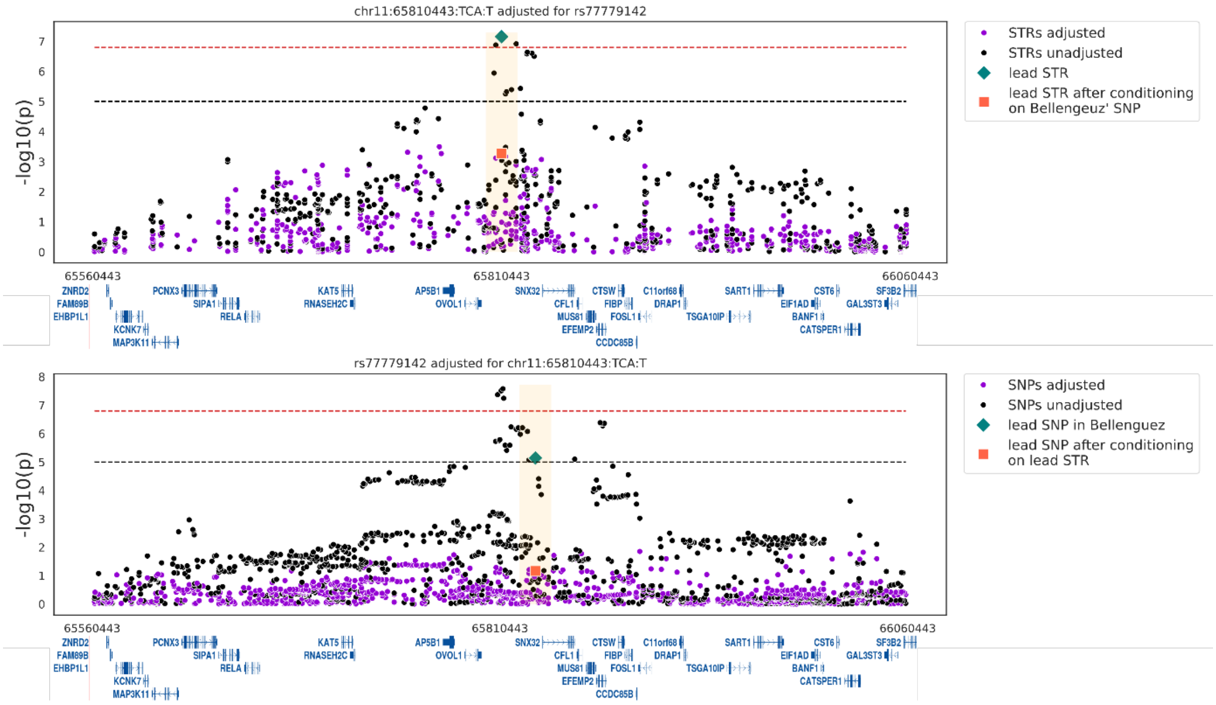

Supplementary Figure 13. STR-SNP pair: chr11:65810443:TCA:T- rs77779142.

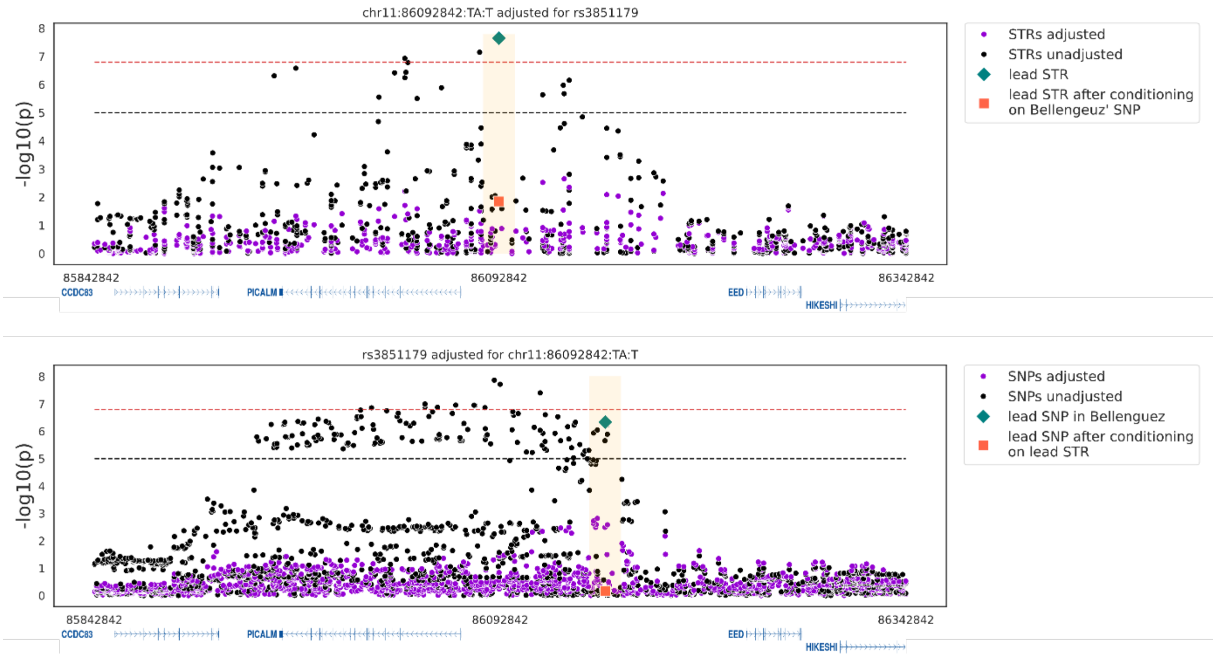

Supplementary Figure 14. STR-SNP pair: chr11:86092842:TA:T - rs3851179.

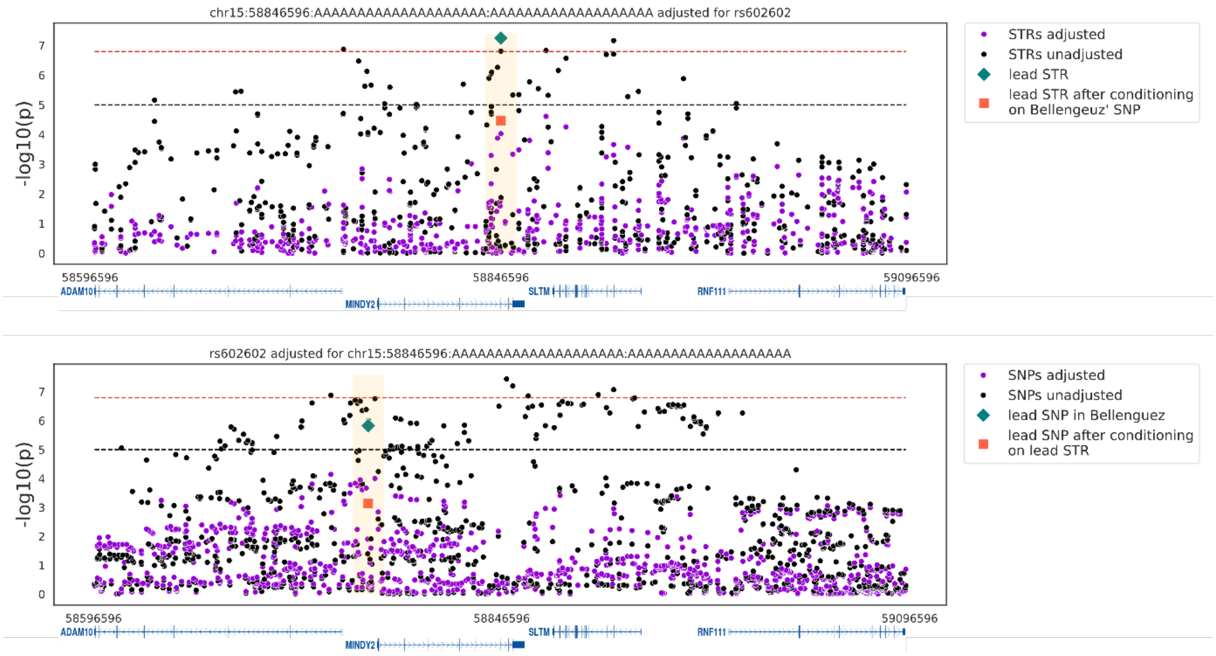

Supplementary Figure 15. STR-SNP pair: chr15:58846596:A20:A19- rs602602.

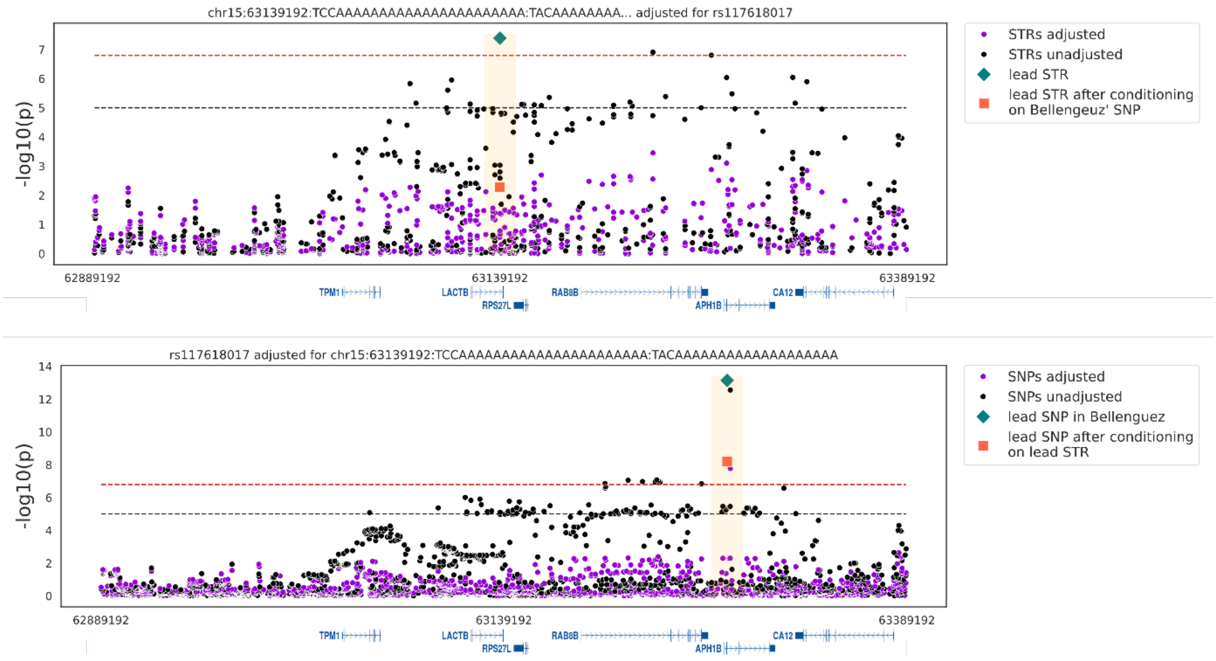

Supplementary Figure 16. STR-SNP pair: chr15:63139192:TCC(A)22:TAC(A)19 - rs117618017.

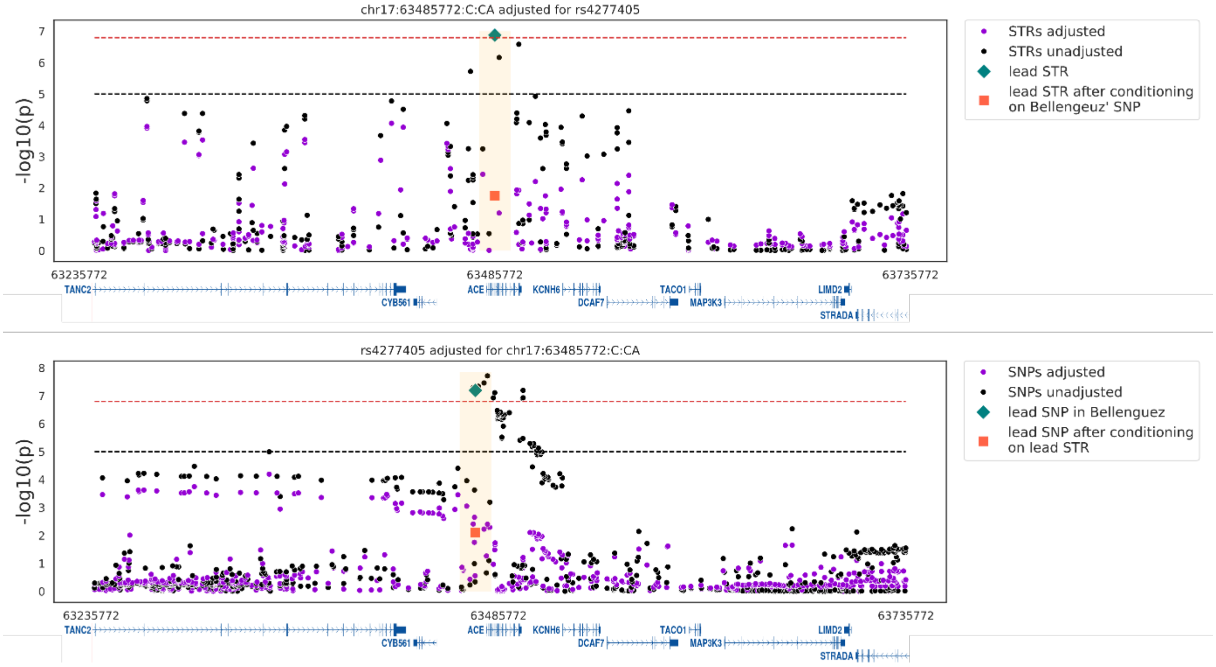

Supplementary Figure 17. STR-SNP pair: chr17:63485772:C:CA - rs4277405.

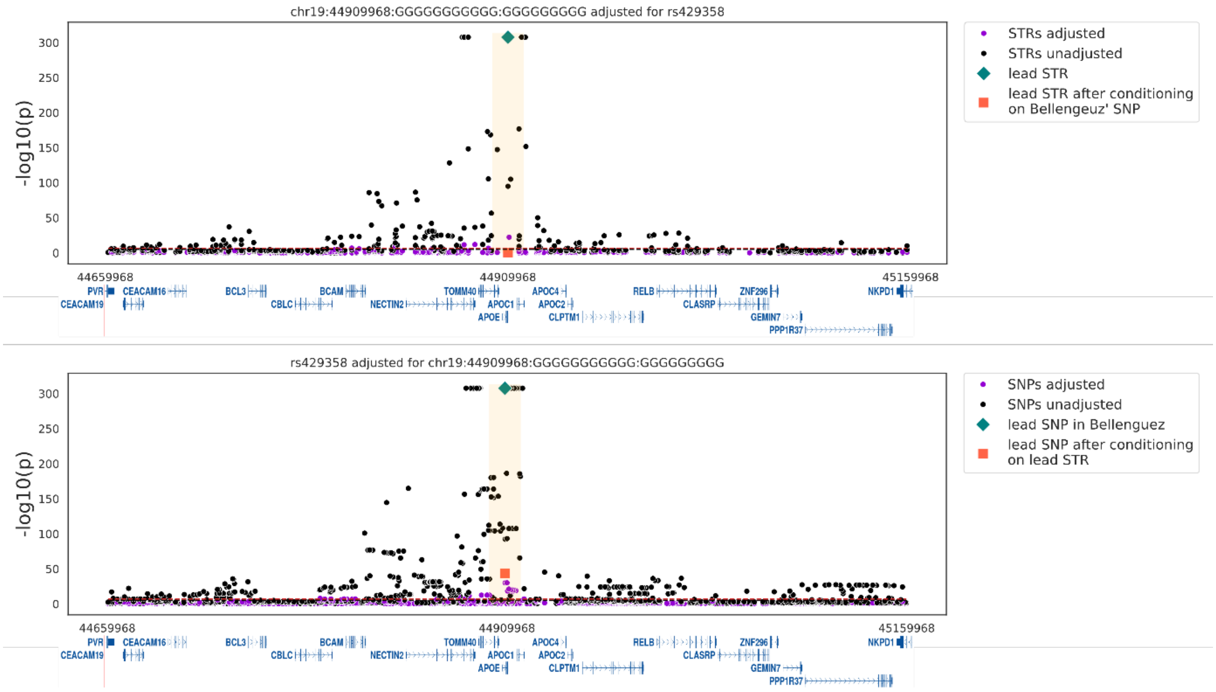

Supplementary Figure 18. STR-SNP pair: chr19:44909968:G11:G9 - rs429358.

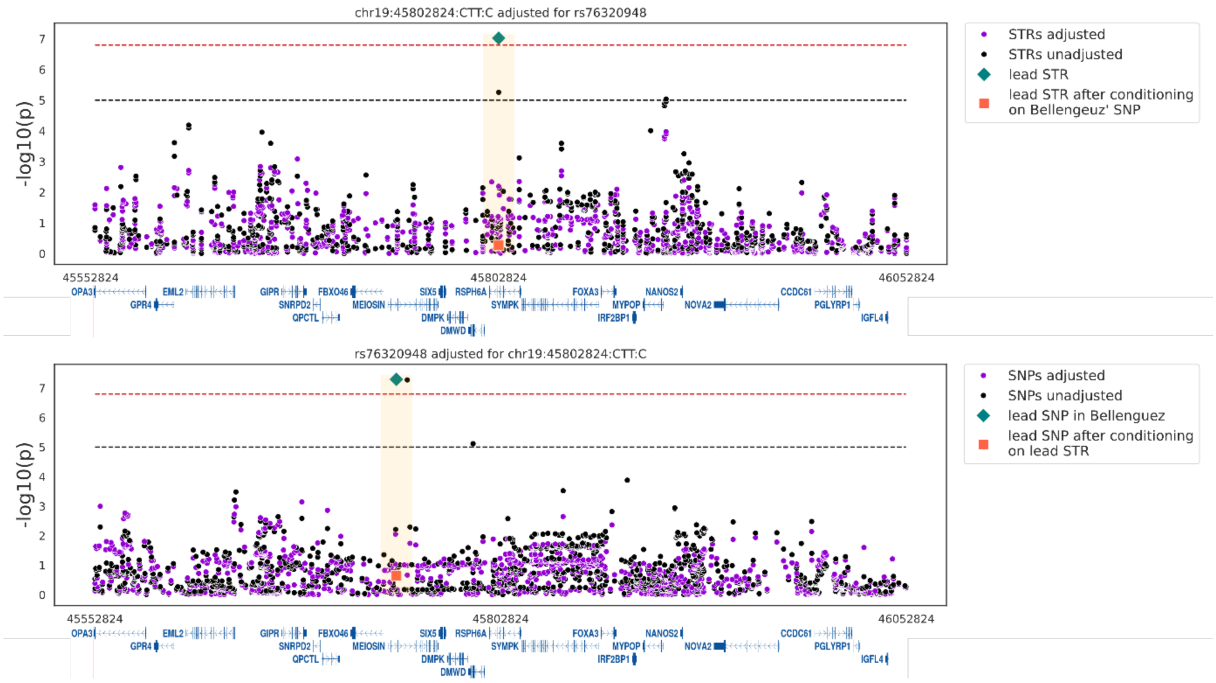

Supplementary Figure 19. STR-SNP pair: chr19:45802824:CTT:C - rs76320948

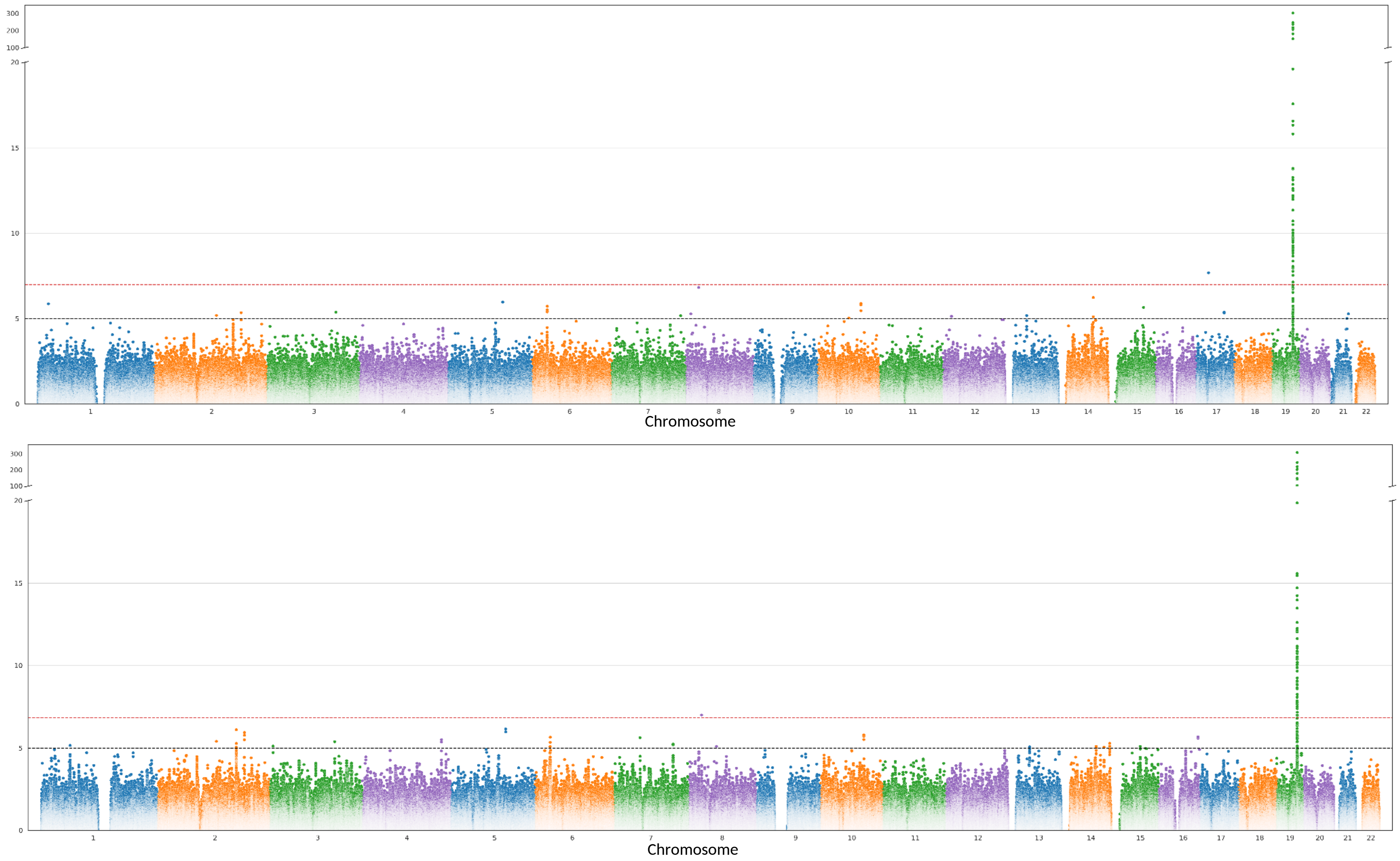

Supplementary Figure 20. Comparison of GWAS results using two-sided linear regression performed on "White-British" subset of samples with WGS data available (n=95,201) using genotyped STRs (upper panel) and imputed STRs (lower panel). The horizontal dashed lines indicate the Bonferroni-corrected genome-wide significance threshold (p<1.01E-07; red) and the suggestive significance threshold (p<1.00E-05; black).

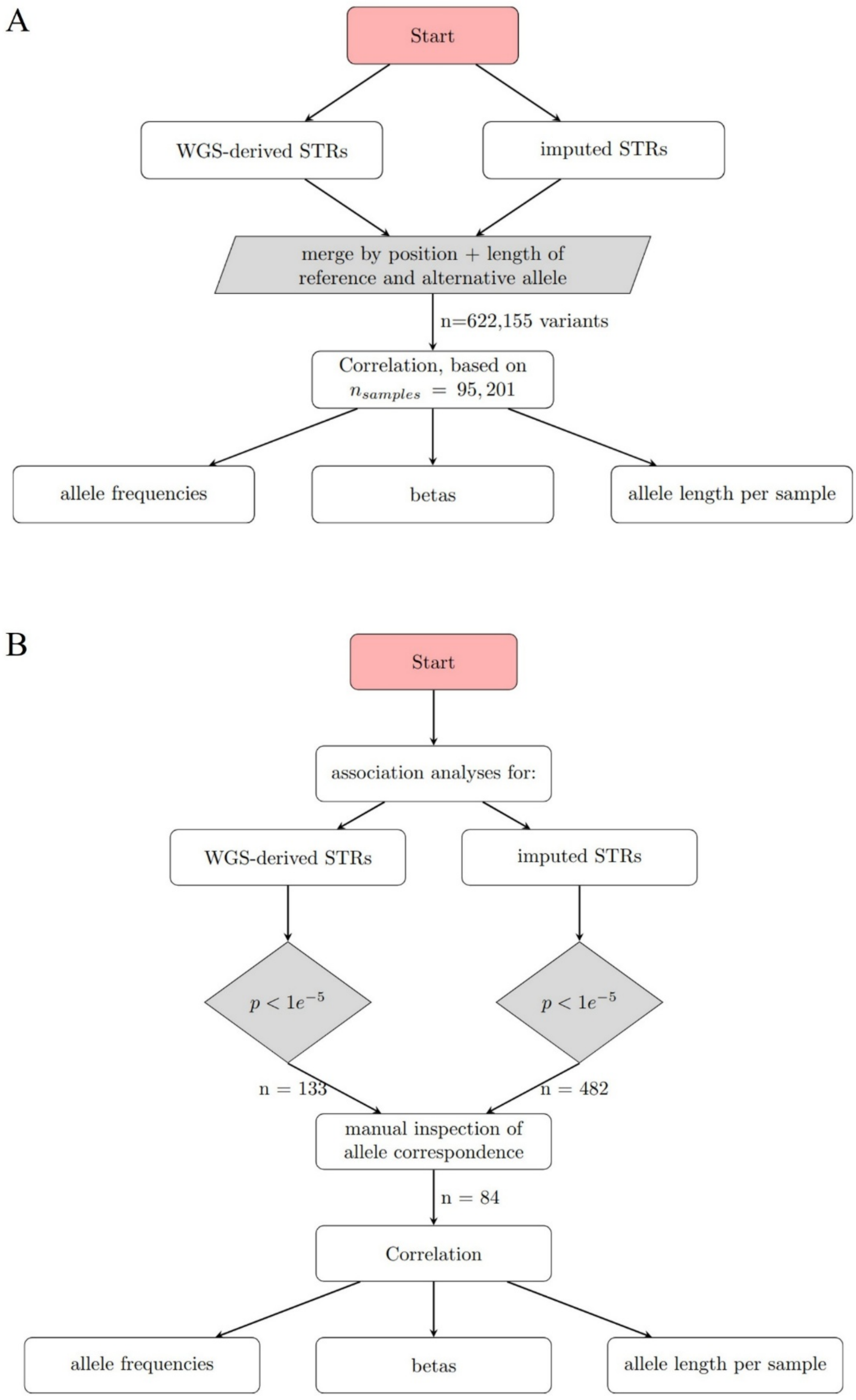

Supplementary Figure 21. Flowcharts of matching procedure between imputed and WGS-derived STRs. (**A**) Matching of STR alleles by genomic position and by allele length (n=622,155). (**B**) Manual curation for variants that minimally passed suggestive significance in the imputed and WGS-derived dataset (matching alleles, lining up positions with UCSC coordinates, checking for SNPs possibly mapping within repeat sequences) (n=84).

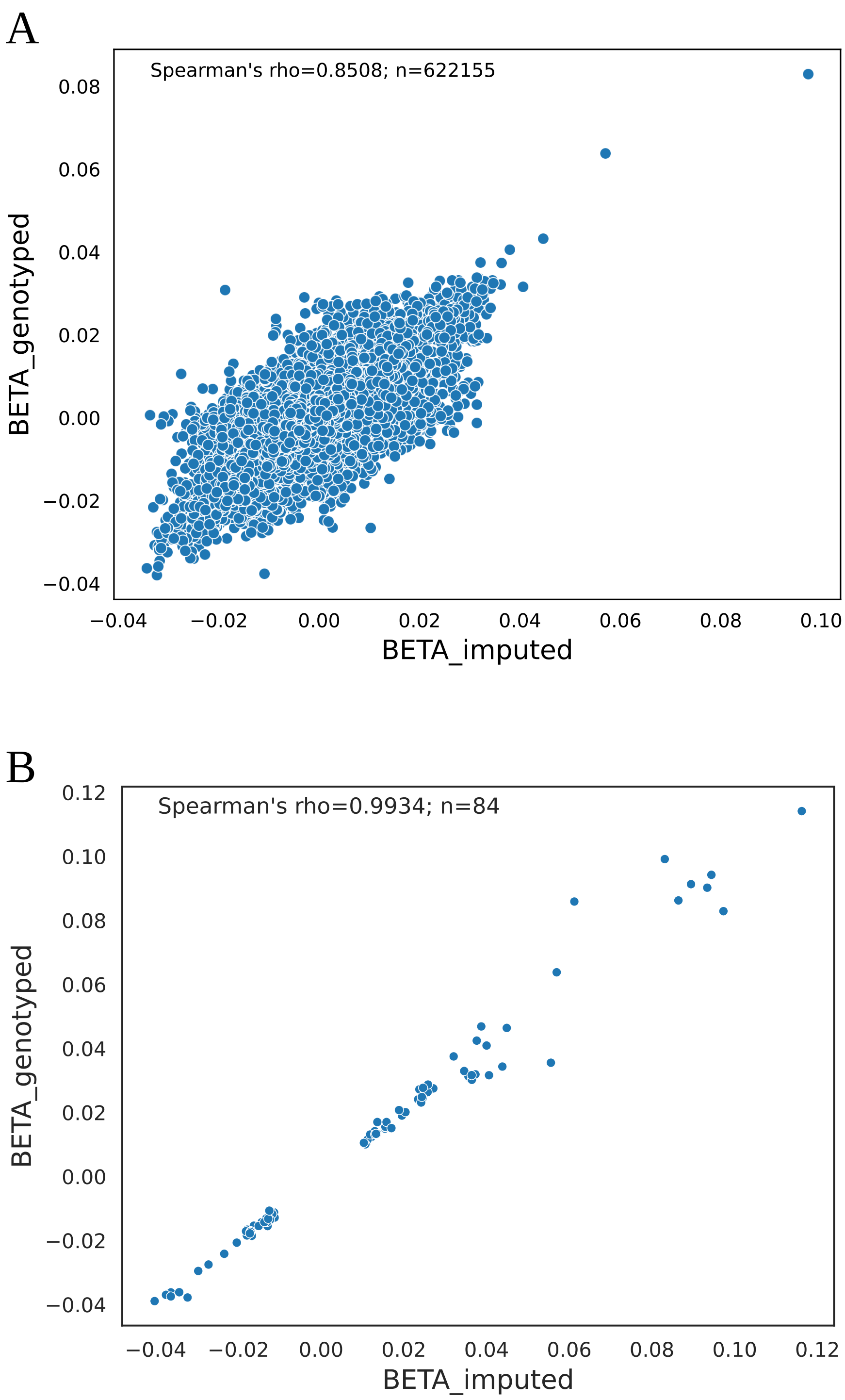

Supplementary Figure 22. Correlation plots of GWAS effect sizes (beta). Correlation coefficient rho was calculated using two-sided Spearman correlation. (**A**) STR variants with unique matches for positions and allele lengths between imputed and WGS-derived STRs (n=622,155, p<2.2E-308). (**B**) The same for STR variants with p<1.0E-05 in both imputed and WGS-derived datasets and manually curated matches (n=84, p=8.77E-79); (see Methods).

A

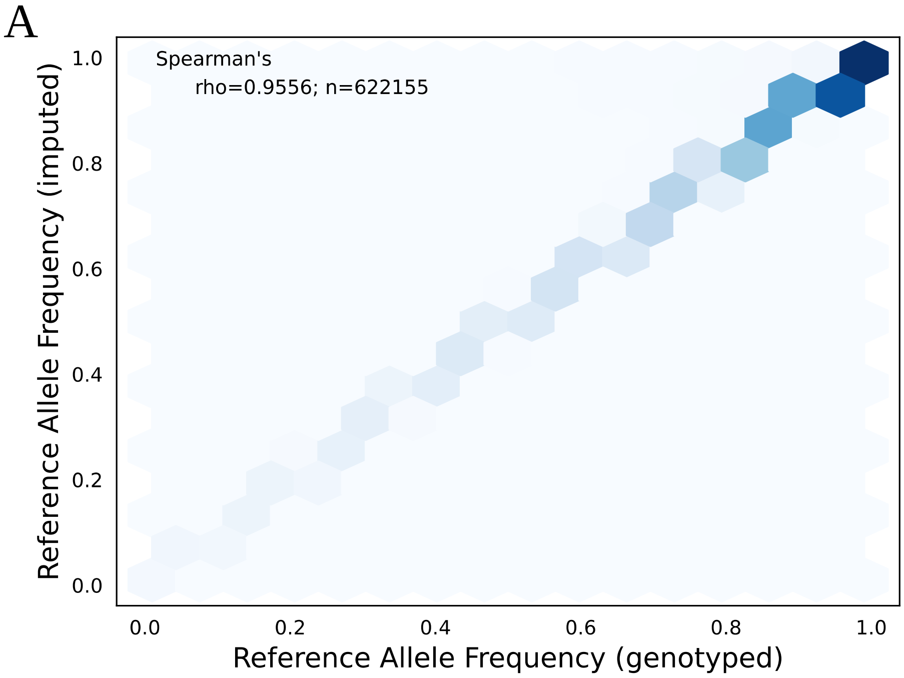

B

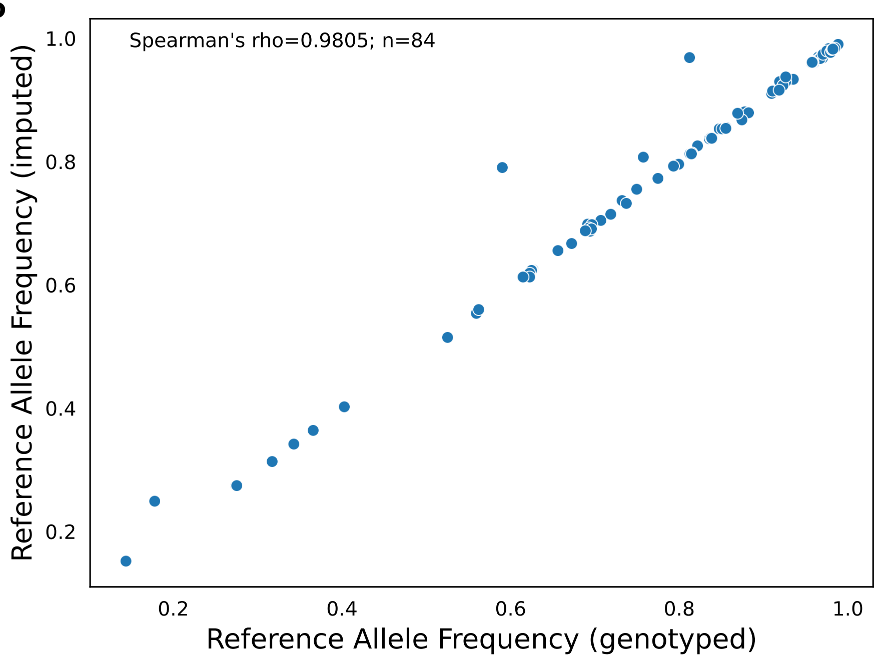

Supplementary Figure 23. Correlation plots of reference allele frequencies. Correlation coefficient rho was calculated using two-sided Spearman correlation. (**A**) Hexbin plot for STR variants with unique matches of positions and allele lengths between imputed and genotyped STRs (n=622,155, p<2.2E-308). Hexbin plot was used since the number of outlying datapoints in 600K dataset is a large number and this aggravates recognition of patterns in regular scatterplots and its subsequent interpretation. The colour gradient represents the number of variants that fall in each region. (**B**) STR variants with p<1.0E-05 in GWAS on both the imputed and genotyped datasets for manually curated matches (n=84, p=1.12e-59); (see Methods).

A

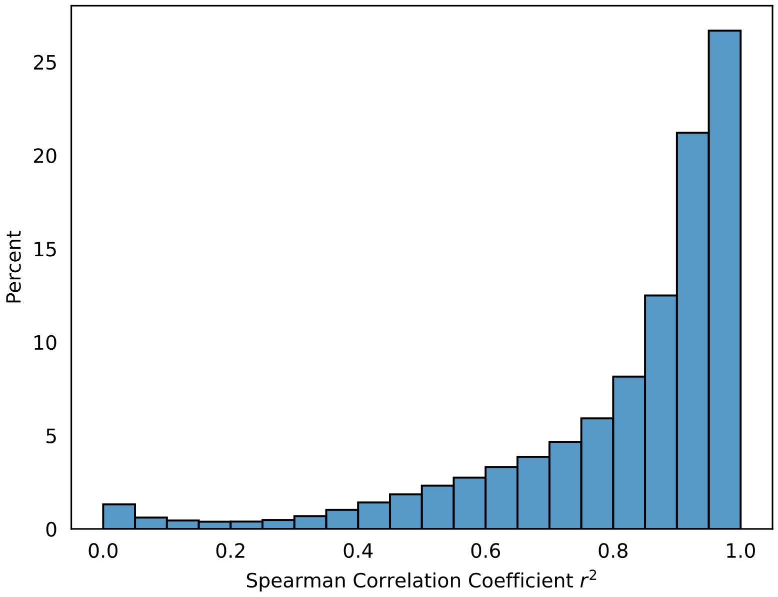

B

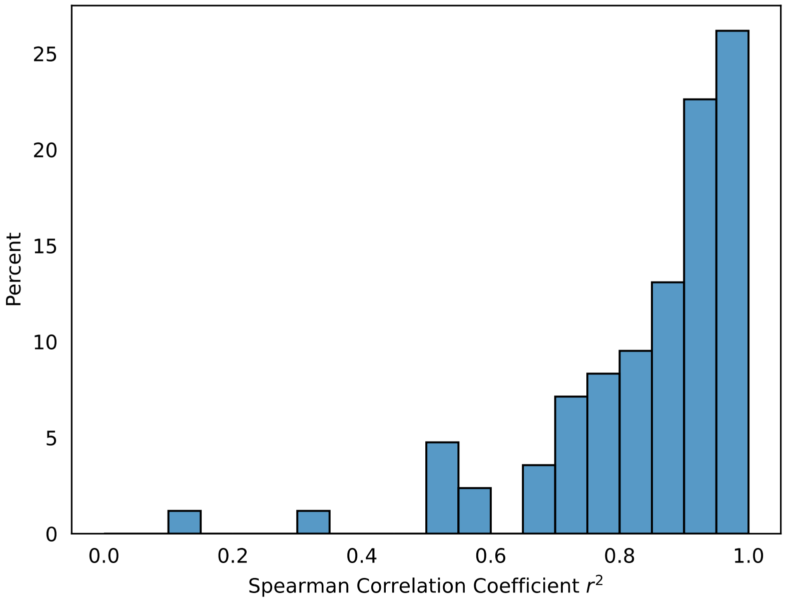

Supplementary Figure 24. Distribution of Spearman correlation coefficients of sum of allele lengths per locus using two-sided tests. (**A**) STR variants with unique matches of positions and allele lengths between imputed and genotyped STRs (n=622,155); (**B**) STR variants with p<1.0E-05 in GWAS on both the imputed and genotyped datasets for manually curated matches (n=84); (see Methods).

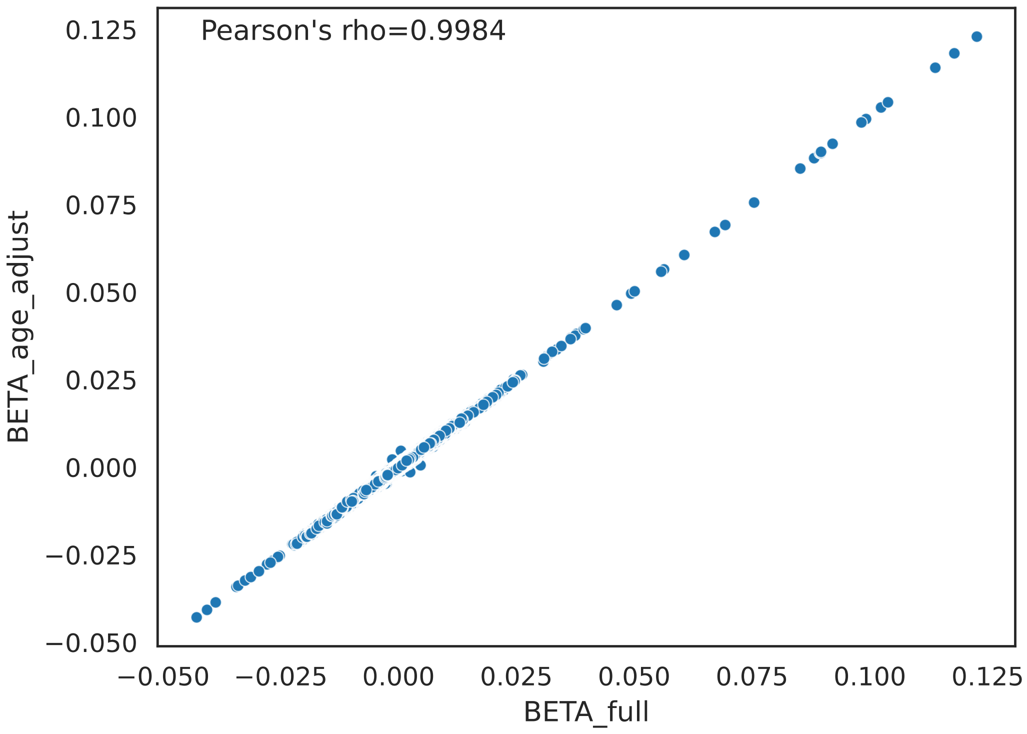

Supplementary Figure 25. Correlation plot of GWAS effect sizes (beta) for association tests with (BETA_age_adjust) vs. without (BETA_full) adjustment for age (n=3,026,383; p<2.2E-308). Correlation coefficient rho was calculated using two-sided Pearson correlation.

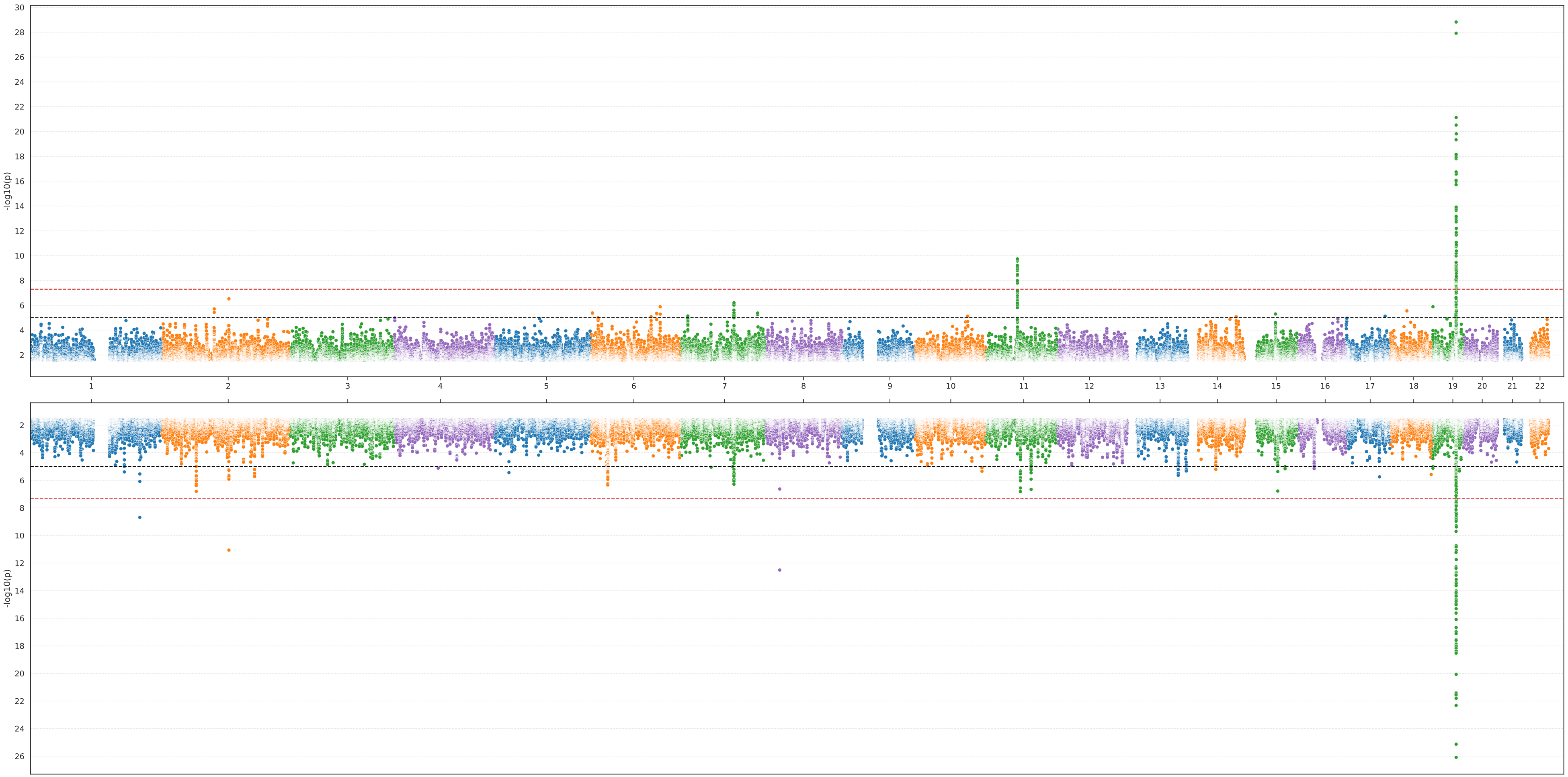

Supplementary Figure 26. Comparison of GWAS results performed on “White-British” subset of samples using two-sided linear regression stratified by sex using imputed STRs. N_male=131,342 (upper panel), N_female=164,209 (lower panel). Horizontal red dashed line indicates the Bonferroni corrected genome-wide significance threshold of 1.49E-07, whereas the black dashed line indicates the suggestive significance threshold of 1.00E-05 (not corrected for multiple comparisons).

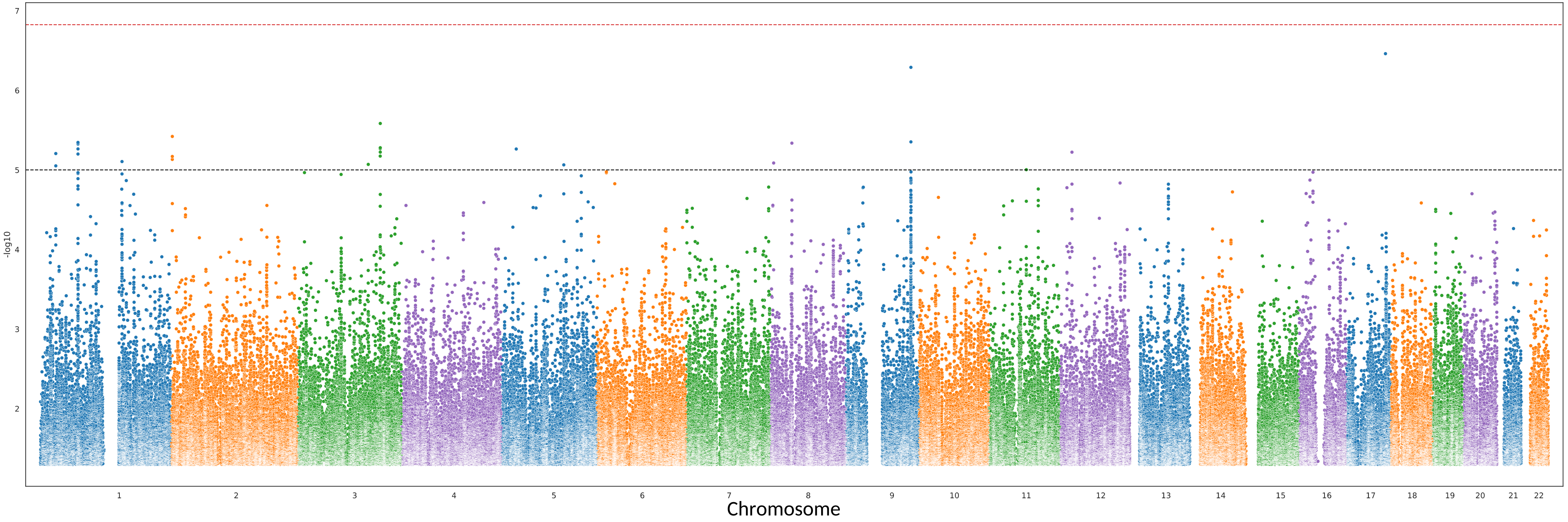

Supplementary Figure 27. Manhattan plot of genome-wide genotype-by-sex (GxS) interaction analysis using two-sided linear regression on "White-British" subset of samples (n=295,551) for imputed STRs. Horizontal red dashed line indicates the Bonferroni-corrected genome-wide significance threshold of 1.49E-07, whereas the black dashed line indicates the suggestive significance threshold of 1.00E-05 (not corrected for multiple testing).

Supplementary References:

1. Bellenguez, C. et al. New insights into the genetic etiology of Alzheimer's disease and related dementias. Nat. Genet. **54**, 412–436 (2022).
2. Jansen, I. E. et al. Genome-wide meta-analysis identifies new loci and functional pathways influencing Alzheimer's disease risk. Nat. Genet. **51**, 404–413 (2019).
