# Supplementary material for: GWAS on short tandem repeats identifies genetic mechanisms in Alzheimer’s disease
Source: Nat Commun. 2026 Jun 4;17:4968. doi: 10.1038/s41467-026-73902-7 (PMC13237113; doi:10.1038/s41467-026-73902-7)
Supplement: Supplementary file 2 — Description of additional supplementary files [file 41467_2026_73902_MOESM2_ESM.pdf]

## Description of Additional Supplementary Files

**File Name:** Supplementary Data 1

**Description: List of unique imputed STRs.** All listed STRs were passing the suggestively significant threshold in GWAS ($p < 1.0E-05$) using two-sided linear regression. Duplicated STRs and non-STR variants were removed. Allele 1 represents the effect allele. Beta and SE are the beta value and the standard error of the estimate in regression t-test.

**File Name:** Supplementary Data 2

**Description: Summary of genome-wide significant GWAS results on imputed STRs.** Association analysis using two-sided linear regression was performed on imputed STRs (bi-allelic approach) in the cohort of self-reported "White-British" individuals from UKB. Allele 1 represents the effect allele. Beta and SE are the beta value and the standard error of the estimate in regression t-test. Gene name refers to the nearest protein-coding gene according to GENCODE V47. Distance from the nearest gene was calculated using the gene coordinates including UTRs. Upstream/downstream designations were assigned with the respect to the gene orientation. As closest known AD-GWAS SNP we used the closest SNP within a ±500kb interval that is listed in Bellenguez et al., 2022 (in Table1 and Table2). However, since the *APOE* region (44–46 Mb on chromosome 19) was excluded in the study by Bellenguez et al., 2022, we used the APOE4 SNP (rs429358) as proxy for STR chr19:44909968:G11:G9 and SNP rs76320948 from Jansen et al., 2019 as proxy for STR chr19:45802824:CTT:C. Of note, STR chr19:1046580:TG:T was nominated as signal-driving by COJO. NA - not available.

**File Name:** Supplementary Data 3

**Description: Summary of suggestively significant GWAS results on imputed STRs.** Association analysis was performed on imputed STRs in self-reported "White-British" individuals from UKB using a two-sided linear regression and bi-allelic approach. Allele 1 represents the effect allele. Beta and SE are the beta value and the standard error of the estimate in regression t-test. Gene name refers to the nearest protein-coding gene according to GENCODE V47. Distance from the nearest gene was calculated using the gene coordinates including UTRs. Upstream/downstream designations were assigned with the respect to the gene orientation. As closest known AD-GWAS SNP we used the closest SNP within a ±500kb interval that is listed in Bellenguez et al., 2022 (in Table1 and Table2). NA - not applicable.

**File Name:** Supplementary Data 4

**Description: Results of multi-allelic association analyses for genome-wide significant top-hits from the biallelic GWAS.** Association analysis was performed on imputed STRs in self-reported "White-British" individuals from UKB. Please note that multi-allelic analyses were limited to variants mapping ±250kb around loci that were at least suggestively significant in the bi-allelic approach. For two STRs (15:63139195 and 19:45802825) corresponding multiallelic STR was not found in the catalogue at the exact position used to label biallelic STR, but alternative starting position was used in the multiallelic approach. Matching was proved in the UCSC database.

**File Name:** Supplementary Data 5

**Description: New lead STR variants emerging from multi-allelic analysis.** Multi-allelic analysis was performed for regions ±250kb around genome-wide and suggestive significant loci from the biallelic

approach (see Supplementary Data 2 and Supplementary Data 3). Only genome-wide significant results are shown. Note that for STR at Chr 14 and 17, there are apparently duplicated entries. This is due to the matching of two different alleles at the same STR in the biallelic approach. NA - not applicable, since the given STR was not found in the biallelic approach. Distance refers to the distance of multiallelic genome-wide significant hit from the top-hit (suggestive or genome-wide significant) in the biallelic approach.

**File Name:** Supplementary Data 6

**Description: Summary of replication analysis and meta-analysis for the genome-wide significant GWAS results.** Replication analysis was performed using a two-sided linear regression on imputed STRs and the "Other White" cohort (n=20,840). Meta-analysis was conducted using fixed-effects model combining the discovery and replication cohort (two-sided linear regression). Discovery cohort consists of UKB participants self-reported as “White-British” ethnicity (n=295,551), while replication cohort included UKB participants self-reported as "Other-White” ethnicity (n=20,840). Allele 1 represents the effect allele. Beta and SE are the beta value and the standard error of the estimate in regression t-test. Gene name refers to the nearest protein-coding gene according to GENCODE V47. NA - not applicable. P value (Meta) is P value of meta-analysis performed across "white" samples. Italicized values indicate stronger associations (by P value) upon fixed effect meta-analysis across the discovery and replication subsets. STRs showing direct evidence of replication in the "Other White" cohort are shown in bold.

**File Name:** Supplementary Data 7

**Description: Summary of SuSiE fine mapping results.** Fine mapping was performed for genome-wide significant GWAS results on imputed STRs in the cohort of self-reported "White-British" individuals from UKB. STR_pip refers to posterior inclusion probability (PIP) for lead STR variant, representing the probability that this variant is causally associated with the trait, as estimated by SuSiE.

Max_locus_pip is the highest PIP observed among all variants in the locus (i.e., 0.5 Mb around our lead STR variant), indicating the most likely causal variant according to the model. Max_source refers to the source (SNP vs STR) of the variant with the highest PIP in the locus (i.e., 0.5 Mb around our lead STR variant). Max_ID is the identifier of the variant with the highest PIP in the locus (i.e., 0.5 Mb around our lead STR variant). Max_r2_to_STR stands for the squared correlation coefficient ($r^2$) to indicate linkage disequilibrium between our lead STR variant and the variant with the highest PIP. Locus_PIP_median refers to the median PIP value across all variants in the locus (i.e., 0.5 Mb around our lead STR variant). Locus_PIP_mean is the mean PIP value across all variants in the locus (i.e., 0.5 Mb around our lead STR variant). Locus_PIP_se is the standard error of the mean PIP across all variants in the locus (i.e., 0.5 Mb around our lead STR variant).

**File Name:** Supplementary Data 8

**Description: Summary of conditioning of genome-wide significant GWAS results.** Conditioning of genome-wide significant GWAS results was performed using two-sided linear regression on imputed STRs in the cohort of self-reported "White-British" individuals from UKB. The Bonferroni correction was applied to adjust for multiple comparisons. Gene name refers to the nearest protein-coding gene according to GENCODE V47. Since the *APOE* region (44–46 Mb on chromosome 19) was excluded in the study by Bellenguez et al., 2022, for chr19:44909968:$G_{11}$:$G_9$, conditioning was performed with the APOE4 SNP, rs429358 (chr19:44908684), and for chr19:45802824:CTT:C, with rs76320948 from Jansen et al., 2019.

**File Name:** Supplementary Data 9

**Description: Imputed STRs that showed stronger evidence for association after conditioning.** Conditioning of the lead STR with respective lead SNP from Bellenguez et al., 2022 was performed

using two-sided linear regression. Allele 1 represents the effect allele. Beta is the beta value of the estimate in regression t-test.

**File Name:** Supplementary Data 10

**Description: Imputed STRs that remained genome wide significant after conditioning with APOE4 SNP.** Conditioning was performed using rs429358 at chr19:44908684 and two-sided linear regression. Allele 1 represents the effect allele. Beta and SE are the beta value and the standard error of the estimate in regression t-test. Values shown in bold indicate that P value changed from non-significant to genome-wide significant after adjusting.

**File Name:** Supplementary Data 11

**Description: Summary of conditioning of suggestive GWAS results.** Conditioning was performed using two-sided linear regression on imputed STRs in the cohort of self-reported "White-British" individuals from UKB. Suggestive GWAS results are defined by an unadjusted threshold of p < 1.00E-05. NA - not available, i.e., conditioning was not performed since there were no lead-SNPs in ±0.5Mb window around this STR in the Bellenguez et al., 2022 study. NA* - not available, since SNP was not available in UKB SNP data.

**File Name:** Supplementary Data 12

**Description: Summary of genome-wide and suggestively significant GWAS results on WGS-derived STRs.** Association analysis was performed on WGS-derived STRs in self-reported "White-British" individuals from UKB using a two-sided linear regression and bi-allelic approach. Allele 1 represents the effect allele. Alternative allele - numbers represent number of repeat units. Beta and SE are the beta value and the standard error of the estimate in regression t-test. Gene name refers to the

nearest protein-coding gene according to GENCODE V47. Distance from the nearest gene was calculated using the gene coordinates including UTRs. Upstream/downstream designations were assigned with the respect to the gene orientation. STRs passing genome-wide suggestive threshold (p=1.01E-07) are shown in bold. For chr19:44921083:M_2, please note that this STR maps 1.7kb downstream from the APOC1 gene. However, due to the well-established role of *APOE* and *APOE* being located only 27kb upstream, this locus is annotated as *APOE*.

**File Name:** Supplementary Data 13

**Description: Summary of conditioning of GWAS results on WGS-derived STRs.** Conditioning analysis was performed for both genome-wide significant and suggestive GWAS results in the cohort of self-reported "White-British" individuals from UKB, using two-sided linear regression. STRs passing genome-wide suggestive threshold (p=1.01E-07) are shown in bold. NA - not applicable, i.e., conditioning was not performed since there were no genome-wide significant SNPs in ±0.5Mb window around this STR in the Bellenguez et al., 2022 study. Since the *APOE* region (44–46 Mb on chromosome 19) was excluded in the study by Bellenguez et al., 2022, for chr19:44921083:M_2, conditioning was performed with the APOE4 SNP, rs429358 (chr19:44908684). For chr17:27264667:M_8, there were no genome-wide significant SNPs within ±0.5Mb region in Bellenguez et al., 2022. Since this was the only genome-wide significant result (apart from *APOE* region), we performed additional conditioning using the suggestively significant SNP within ±0.5Mb with the lowest P value that was available in the summary statistics supplied by Bellenguez et al., 2022 (rs117222268).

**File Name:** Supplementary Data 14

**Description: Significant results of meQTL analysis for genome-wide significant UKB STR-GWAS signals.** meQTL analysis was performed using two-sided linear regression. To adjust for multiple

comparisons results Benjamini-Hochberg procedure was applied (FDR <5%). Gene name refers to the nearest protein-coding gene according to GENCODE V47. Distance from the nearest gene was calculated using the gene coordinates including UTRs. Upstream/downstream designations were assigned with the respect to the gene orientation. If CpG maps in lncRNA, its ID is given in square brackets. Beta value refers to the reference allele. Please note that chr6:32611487:TCTTTC:T represent alternative allelic variant at the same STR locus that is given in the preceding line (chr6:32611487:TCTTTCTTTC:T). CpGs associated with exactly the same STR allele that was also associated with AD risk are shown in bold. Functional elements are based on the ENCODE Registry of candidate cis-Regulatory Elements (cCREs) in the human genome.

**File Name:** Supplementary Data 15

**Description: Significant results of eQTL analysis for genome-wide significant UKB STR-GWAS signals.** eQTL analysis was performed using two-sided linear regression. To adjust for multiple comparisons results Benjamini-Hochberg procedure was applied (FDR <5%). Gene name refers to the nearest protein-coding gene according to GENCODE V47.

**File Name:** Supplementary Data 16

**Description: Significant results of eQTM analysis for CpGs emerging from meQTL analysis.** eQTM analysis was performed using two-sided linear regression for CpGs emerging from meQTL analysis (Supplementary Data 14). We considered results passing unadjusted threshold of $p < 0.05$ to be significant. Correlation coefficient R is determined using Pearson correlation.

**File Name:** Supplementary Data 17

**Description: Summary of GWAS results on imputed STRs with and without adjusting for age as a covariate.** Association analysis was performed on imputed STRs in the cohort of self-reported "White-British" individuals from UKB using a two-sided linear regression and bi-allelic approach. STRs that show the lowest P value and show at least genome-wide significance (adjusted for multiple comparisons using Bonferroni correction) in either approach are shown. The lead variant is defined as the variant with the lowest P value within a 250kb window for loci passing genome-wide significance.

**File Name:** Supplementary Data 18

**Description: Summary of sex-stratified and sex-interaction GWAS results on imputed STRs.** Association analysis was performed on imputed STRs in the cohort of self-reported "White-British" individuals from UKB using a two-sided linear regression and bi-allelic approach. Only variants reaching genome-wide significance (adjusted for multiple comparisons using Bonferroni correction) in at least one analysis are shown. The lead variant is defined as the variant with the lowest P value within a 250kb window for loci passing genome-wide significance in the respective cohort.

**File Name:** Supplementary Data 19

**Description: Summary of WGS-derived STRs before and after adjusting with APOE4**. Conditioning was performed with the APOE4 SNP (rs429358 at chr19:44908684) using two-sided linear regression. Variants reaching genome-wide significance (adjusted for multiple comparisons using Bonferroni correction) in either analysis are shown. P values that changed from non-significant to genome-wide significant after adjusting are shown in bold.
