# Supplementary material for: GWAS on short tandem repeats identifies genetic mechanisms in Alzheimer’s disease
Source: Nat Commun. 2026 Jun 4;17:4968. doi: 10.1038/s41467-026-73902-7 (PMC13237113; doi:10.1038/s41467-026-73902-7)
Supplement: Supplementary file 22 — Reporting Summary [file 41467_2026_73902_MOESM22_ESM.pdf]

nature portfolio

Corresponding author(s): Lars Bertram

Last updated by author(s): May 12, 2026

Reporting Summary

Nature Portfolio wishes to improve the reproducibility of the work that we publish. This form provides structure for consistency and transparency in reporting. For further information on Nature Portfolio policies, see our Editorial Policies and the Editorial Policy Checklist.

Statistics

For all statistical analyses, confirm that the following items are present in the figure legend, table legend, main text, or Methods section.

n/a	Confirmed	
☐	☒	The exact sample size (*n*) for each experimental group/condition, given as a discrete number and unit of measurement
☐	☒	A statement on whether measurements were taken from distinct samples or whether the same sample was measured repeatedly
☐	☒	The statistical test(s) used AND whether they are one- or two-sided<br>*Only common tests should be described solely by name; describe more complex techniques in the Methods section.*
☐	☒	A description of all covariates tested
☐	☒	A description of any assumptions or corrections, such as tests of normality and adjustment for multiple comparisons
☐	☒	A full description of the statistical parameters including central tendency (e.g. means) or other basic estimates (e.g. regression coefficient) AND variation (e.g. standard deviation) or associated estimates of uncertainty (e.g. confidence intervals)
☐	☒	For null hypothesis testing, the test statistic (e.g. *F*, *t*, *r*) with confidence intervals, effect sizes, degrees of freedom and *P* value noted<br>*Give P values as exact values whenever suitable.*
☒	☐	For Bayesian analysis, information on the choice of priors and Markov chain Monte Carlo settings
☒	☐	For hierarchical and complex designs, identification of the appropriate level for tests and full reporting of outcomes
☐	☒	Estimates of effect sizes (e.g. Cohen's *d*, Pearson's *r*), indicating how they were calculated

Our web collection on statistics for biologists contains articles on many of the points above.

Software and code

Policy information about availability of computer code

Data collection	Early QC steps before imputation were performed using PLINK v2.00a3. For Liftover we used GATK LivtoverVCF (V 4.1.9.0). For imputation we used Conform-GT (Version 24May16) and Beagle (V5.4.). After imputation, initial QC steps were done using bcftools (V. 1.21) and vcftools (V. 0.1.16).
Data analysis	No new software was developed for the analyses of this project. Instead, all software tools were previously published.GWAS QC steps and association analyses were run using PLINKv2.00a3LM. For Meta analysis of GWAS results we used PLINK v1.90b7.Conditional analyses were performed using GCTA-COJO and hertability analyses using GCTA-LDMS, both using GCTA-Version 1.92.0. For meQTL and eQTL analysis we used the R-package MatrixEQTL (V. 2.3), eQTM analyses were run using base packages in R (V 4.4.0 throughout). Finemapping with susieR (V. 0.14.2) was done in R (V.4.3.3). For Spearmans rank correlation and FDR-correction we used the Python packages SciPy (V. 1.13.0) and statsmodels (0.14.4), respectively. For large scale evaluation (e.g. ranking, comparison, plots, replication) we used python packages pandas (1.5.3), numpy (1.25.2), seaborn (0.13.2), matplotlib (3.7.1).

For manuscripts utilizing custom algorithms or software that are central to the research but not yet described in published literature, software must be made available to editors and reviewers. We strongly encourage code deposition in a community repository (e.g. GitHub). See the Nature Portfolio guidelines for submitting code & software for further information.

Data

Policy information about availability of data

All manuscripts must include a data availability statement. This statement should provide the following information, where applicable:

- Accession codes, unique identifiers, or web links for publicly available datasets
- A description of any restrictions on data availability
- For clinical datasets or third party data, please ensure that the statement adheres to our policy

Individual-level genetic and phenotypic data used in this study are available under restricted access due to the legal terms of the UK Biobank Material Transfer Agreement from the UK Biobank (https://www.ukbiobank.ac.uk/enable-your-research/register). Access to the UK Biobank can be obtained by bona fide researchers. The individual-level imputed STR data generated in this study will be returned to the UK Biobank. Access can be obtained by approved researchers through the UK Biobank as a 'Returned Dataset' (under application ID 81874) once UKB's internal processing is complete. The raw individual-level genetic data are protected and are not publicly available due to data privacy laws. The SNP-STR imputation panel is available in the Zenodo database under accession code 10.5281/zenodo.8365671 [https://doi.org/10.5281/zenodo.8365671]. Access to the OPTIMA dataset is restricted due to data protection regulations. Principal investigators can be contacted with data access requests via their research group portal (https://www.pharm.ox.ac.uk/research/groups/smith-group-oxford-project-to-investigate-memory-and-ageing-optima-and-b-vitamin-research-group), subject to the execution of a standard data use agreement.
GWAS summary statistics generated in this study have been deposited in the Zenodo database under accession code 10.5281/zenodo.17908176 [https://doi.org/10.5281/zenodo.17908176].
The previously published GWAS summary statistics from Bellenguez et al.3 used in this study are available in the GWAS Catalog database under accession code GCST90027158 [https://www.ebi.ac.uk/gwas/publications/35379992]. Summary statistics from Jansen et al.24 have been retrieved from DOI 10.1038/s41588-018-0311-9 [https://doi.org/10.1038/s41588-018-0311-9].

Research involving human participants, their data, or biological material

Policy information about studies with human participants or human data. See also policy information about sex, gender (identity/presentation), and sexual orientation and race, ethnicity and racism.

Reporting on sex and gender	The sex of participants (both genotyped and self-reported) was provided by UK Biobank. Samples with mismatches in genetic and reported sex were removed from all downstream analysis. Sex distributions are reported in our study. Analytically, sex is used as a covariate in main GWAS analyses. Additionally, we performed sex-stratified GWAS and a genome-wide interaction analysis using "genotype x sex". Association and QTL analyses were adjusted for sex.
Reporting on race, ethnicity, or other socially relevant groupings	Samples are split into reported ethnic group at baseline visit. This information is provided by the UKB (UKB Field ID 21000).
Population characteristics	For the main study we used UK Biobank samples. The UKB recruited ~500.000 mostly healthy 40–69 old volunteers from 2006 to 2010, which makes them ~55-89 old today. Over 90% of samples were of "white" ethnicity. UKB participants are less likely to live in socioeconomically deprived areas and have a more healthy lifestyle than the general population.
Recruitment	Participants are recruited by the UKB where they provide detailed information using screen questionnaires and provide blood, urine and saliva samples. There is evidence of a participation bias in the UKB, of more healthy or more health-conscious volunteers.
Ethics oversight	The study design and conduct complied with all relevant regulations regarding the use of human study participants. Collection of the UKB data was approved by the Research Ethics Committee of the UKB obtained under application ID 81874. For the Optima Dataset, the Ethics Committees of Oxford University and the University of Lübeck approved the use of the human tissues. The overall study was conducted in accordance with the Declaration of Helsinki, and participants for both the UKB and OPTIMA data gave informed consent.

Note that full information on the approval of the study protocol must also be provided in the manuscript.

Field-specific reporting

Please select the one below that is the best fit for your research. If you are not sure, read the appropriate sections before making your selection.

☒ Life sciences ☐ Behavioural & social sciences ☐ Ecological, evolutionary & environmental sciences

For a reference copy of the document with all sections, see nature.com/documents/nr-reporting-summary-flat.pdf

Life sciences study design

All studies must disclose on these points even when the disclosure is negative.

Sample size	We used all samples that were available to us after QC (n=333,446) and split them according to reported ethnic group. All samples that are of self-reported 'white-british' ethnicity (n=295,551) were considered to be our discovery-dataset. Samples that identify as 'white' but not as 'white-british' are considered to be replication data. For heritability analysis we only used ICD-diagnosed AD cases (n=2,947) and 15,000 randomly selected controls.

Data exclusions	We excluded n=1,805 samples with mismatches in reported and genotyped sex, aneuploidy, or heterozygosity outliers. We also excluded non-cases that do not provide reliable information about parental age and health status, since this is required for proxy-status calculation. This information does not need to be specified for AD cases, as these do not require a proxy status.<br>We removed 169 samples with 10 or more relatives identified by the UKB and ran kinship removal tests prioritizing cases and proxy-cases above controls and removed additional 59,447 individuals.<br>For the second arm of our analysis that is based on WGS-derived STRs, we checked remaining samples for availability of WGS-derived STR genotypes, leaving 107,289 samples.<br>Variants were filtered for minor allele frequency and genotyping efficiency (maf 0.01 –geno 0.02) and variants with deviations from HWE (–hwe 5e-06) in controls.
Replication	We performed replication analysis using a dedicated replication dataset as described above. Not all attempts of replication were successful: Of our main GWAS analysis 12 out of 14 identified genome-wide significant variants showed some either direct or indirect evidence for replication. Of these, six loci also showed at least nominal significance ($p<0.05$).
Randomization	We did not perform any randomization as this is not needed nor informative for this type of study (GWAS).
Blinding	Blinding was not relevant during data collection, as we utilized a pre-existing database with independent, automated genotyping. During data analysis, investigator blinding was not applicable because all quality control measures and statistical association tests were executed using standardized, computational tools with pre-defined statistical thresholds.

Reporting for specific materials, systems and methods

We require information from authors about some types of materials, experimental systems and methods used in many studies. Here, indicate whether each material, system or method listed is relevant to your study. If you are not sure if a list item applies to your research, read the appropriate section before selecting a response.

Materials & experimental systems

n/a	Involved in the study	
☒	☐	Antibodies
☒	☐	Eukaryotic cell lines
☒	☐	Palaeontology and archaeology
☒	☐	Animals and other organisms
☒	☐	Clinical data
☒	☐	Dual use research of concern
☒	☐	Plants

Methods

n/a	Involved in the study	
☒	☐	ChIP-seq
☒	☐	Flow cytometry
☒	☐	MRI-based neuroimaging

Plants

Seed stocks	*Report on the source of all seed stocks or other plant material used. If applicable, state the seed stock centre and catalogue number. If plant specimens were collected from the field, describe the collection location, date and sampling procedures.*
Novel plant genotypes	*Describe the methods by which all novel plant genotypes were produced. This includes those generated by transgenic approaches, gene editing, chemical/radiation-based mutagenesis and hybridization. For transgenic lines, describe the transformation method, the number of independent lines analyzed and the generation upon which experiments were performed. For gene-edited lines, describe the editor used, the endogenous sequence targeted for editing, the targeting guide RNA sequence (if applicable) and how the editor was applied.*
Authentication	*Describe any authentication procedures for each seed stock used or novel genotype generated. Describe any experiments used to assess the effect of a mutation and, where applicable, how potential secondary effects (e.g. second site T-DNA insertions, mosiacism, off-target gene editing) were examined.*
